# Supplementary material for: STING-dependent sensing of self-DNA drives silica-induced lung inflammation
Source: Nat Commun. 2018 Dec 6;9:5226. doi: 10.1038/s41467-018-07425-1 (PMC6283886; doi:10.1038/s41467-018-07425-1)

Supplementary Information

STING-dependent sensing of self-DNA drives silica-induced lung inflammation

Benmerzoug et al.

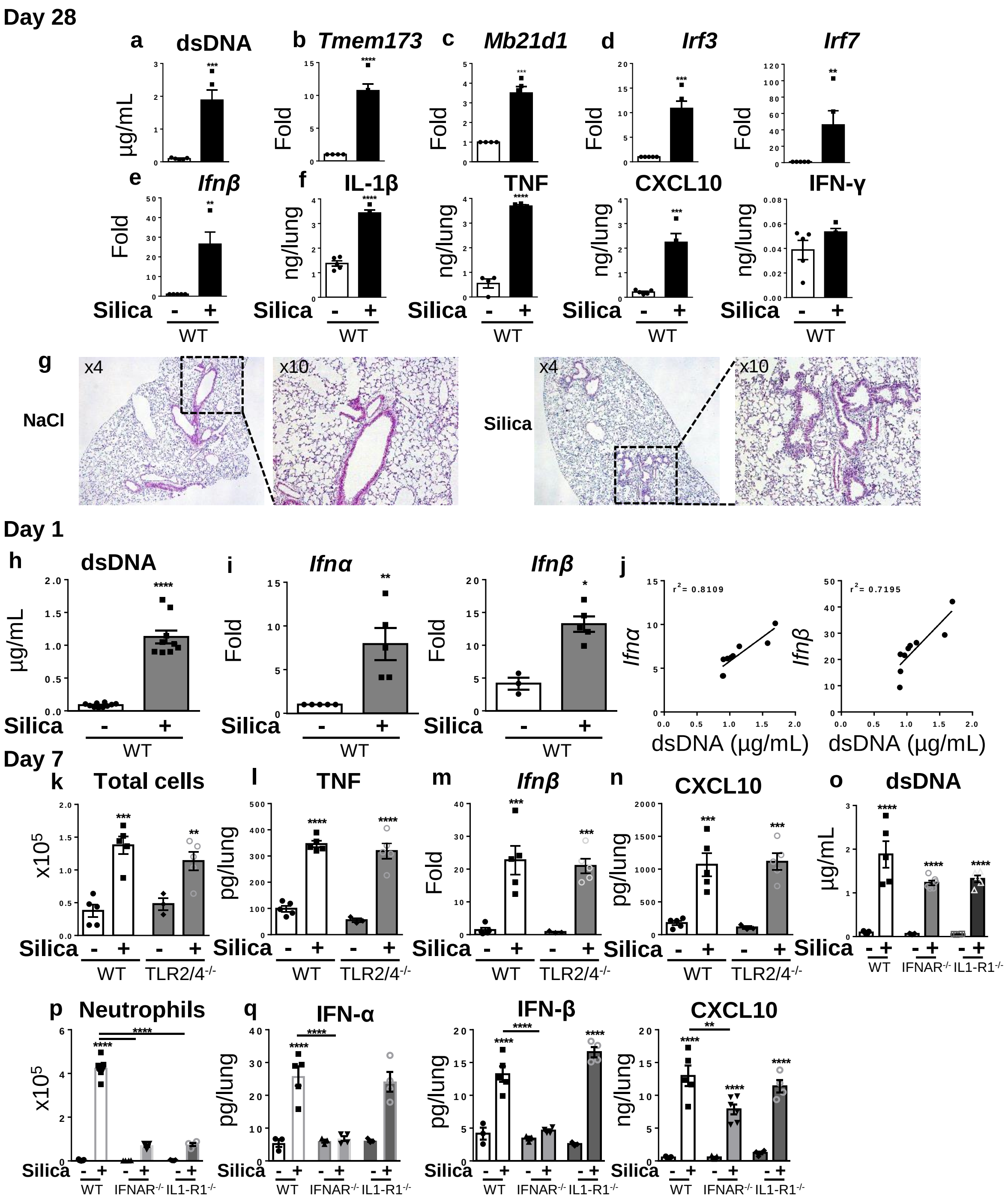
Day 28
a dsDNA
b Tmem173
c Mb21d1
d Irf3
Irf7
e Ifnβ
f IL-1β
TNF
CXCL10
IFN-γ
Silica - +
WT
g
NaCl
Silica
x4
x10
Day 1
h dsDNA
i Ifnα
Ifnβ
j
r² = 0.8109
r² = 0.7195
dsDNA (μg/mL)
Day 7
k Total cells
l TNF
m Ifnβ
n CXCL10
o dsDNA
WT
TLR2/4-/-
IFNAR-/-
IL1-R1-/-
p Neutrophils
q IFN-α
IFN-β
CXCL10

Supplementary Figure 1 – Self-dsDNA release and type I IFN dependent lung inflammation in acute and chronic silica-airway exposure models.

(a-g) Silica microparticles (1 mg/mouse i.t.) or saline vehicle were administered to WT mice and the different parameters analysed on day 28 post-exposure. (a) Concentration of extracellular dsDNA in the bronchoalveolar lavage fluid (BALF) acellular fraction. (b) *Tmem173,* (c) *Mb21d1,* (d) *Irf3* and *Irf7* and (e) *Ifnβ* transcripts measured in the lung by real-time PCR. (f) Lung levels of IL-1β, TNF, CXCL10 and IFN-γ quantified by ELISA. (g) Lung HE staining at day 28 post-silica exposure (4 and 10X magnification).

(h-j) Silica microparticles or saline vehicle were administered to WT mice as in (a) and parameters analysed on day 1 post-exposure. (h) Concentration of extracellular dsDNA in the BALF acellular fraction. (i) *Ifnα* and *Ifnβ* transcripts were evaluated by real-time PCR and (j) correlated with self-dsDNA release in the BALF.

(k-n) Silica microparticles (1mg/mouse i.t.) or saline vehicle as in (a) were administered to WT and TLR2/TLR4$^{-/-}$ mice and parameters analyzed on day 7 post-exposure. (k) Total cells in the BAL. (l) Lung levels of TNF measured by ELISA, (m) *Ifnβ* transcripts measured by real-time PCR, and (n) CXCL10 measured by ELISA.

(o-q) Silica microparticles or saline vehicle as in (a) were administered to WT, IFNAR$^{-/-}$ and IL-1R1$^{-/-}$ mice and parameters analyzed on day 7 post-exposure. (o) Concentration of extracellular dsDNA in the BALF. (p) Neutrophils counts in the BALF. (q) Lung levels of IFN-α, IFN-β and CXCL10 measured by multiplex immunoassay and ELISA, respectively.

*p < 0.05, **p < 0.01, ***p < 0.001, ****p < 0.0001 (Student's t test (a-i) or Kruskal-Wallis test followed by Dunn post-test (k-p)). The correlation analysis were nonparametric (Spearman's correlation). Each symbol represents an individual mouse. Data are representative of two independent experiments (a-i: mice per group: n= 3 or 5 (WT NaCl), n= 5 or 9 (WT Silica) or are representative of at least two independent experiments with similar results; k-n: mice per group: n= 5 (WT NaCl), n= 5 (WT Silica), n= 3 (TLR2/4$^{-/-}$ NaCl), n= 5 (TLR2/4$^{-/-}$ Silica); o-p: mice per group: n= 3 (WT NaCl), n= 5 (WT Silica), n= 3 (IFNAR$^{-/-}$ NaCl), n= 4 or 6 (IFNAR$^{-/-}$ Silica), n= 3 (IL1-R1$^{-/-}$ NaCl), n= 4 (IL1-R1$^{-/-}$ Silica)). Source data are provided as a Source Data file.

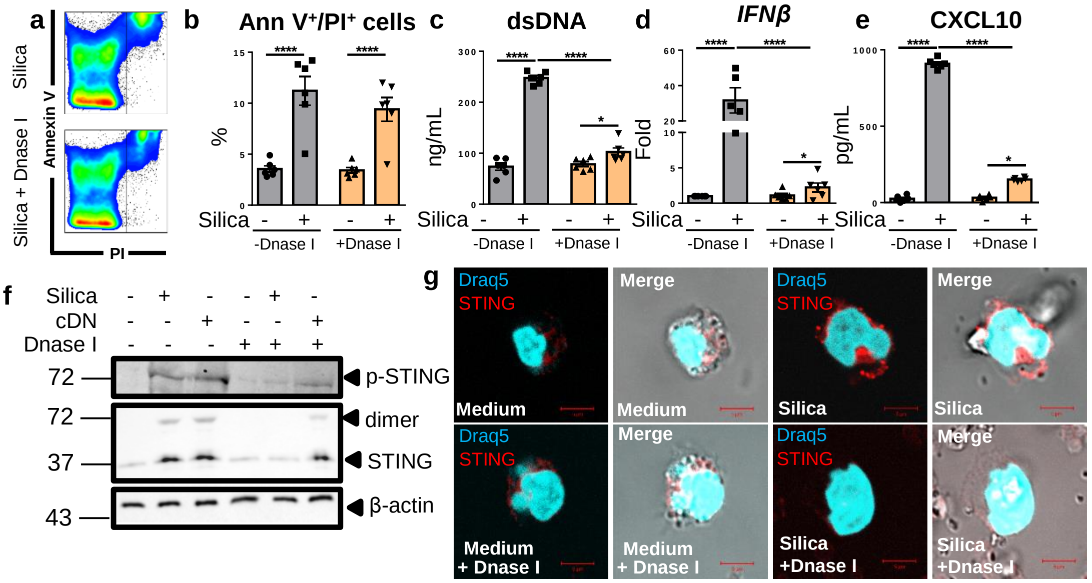

Supplementary Figure 2. Degradation of extracellular self-dsDNA abrogates STING-mediated type I IFN response in silica-exposed human PBMCs.

Extracellular DNase I treatment (1 µg/mL) was applied 3 hr prior and 1 hr after silica exposure (250 µg/mL) or transfection with c-di-AMP (6 µg/mL; cDN) for 18 hr in human PBMCs.

(a) Flow cytometry analysis of Annexin V/PI staining showing early apoptosis (Ann V^+/PI^-) versus late apoptosis/necrosis (Ann V^+/PI^+) in human PBMCs (gated on singlet cells).

(b) Proportion of Annexin V^+/PI^+ human PBMCs.

(c) Concentration of extracellular dsDNA in PBMC supernatant.

(d) Level of *IFNβ* transcript determined by RT-qPCR and normalized to *GAPDH* expression.

(e) Concentration of CXCL10 in PBMC supernatant measured by ELISA.

(f) Immunoblots of phosphorylated STING (p-STING), STING on human PBMCs, with β-actin as a reference.

(g) Confocal Images of human PBMCs stained with DNA dye Draq5 (cyan) and STING specific antibody (red). Bars, 5µM.

*p < 0.05, **p < 0.01, ***p < 0.001, ****p < 0.0001 (Kruskal-Wallis test followed by Dunn post-test). Each symbol represents an individual. Data are presented as mean ± SEM and are representative of two independent experiments. (a-e: patients per group: n= 6 or are representative of n= 6 samples from at least two independent experiments with similar results (f-g)). Source data are provided as a Source Data file.

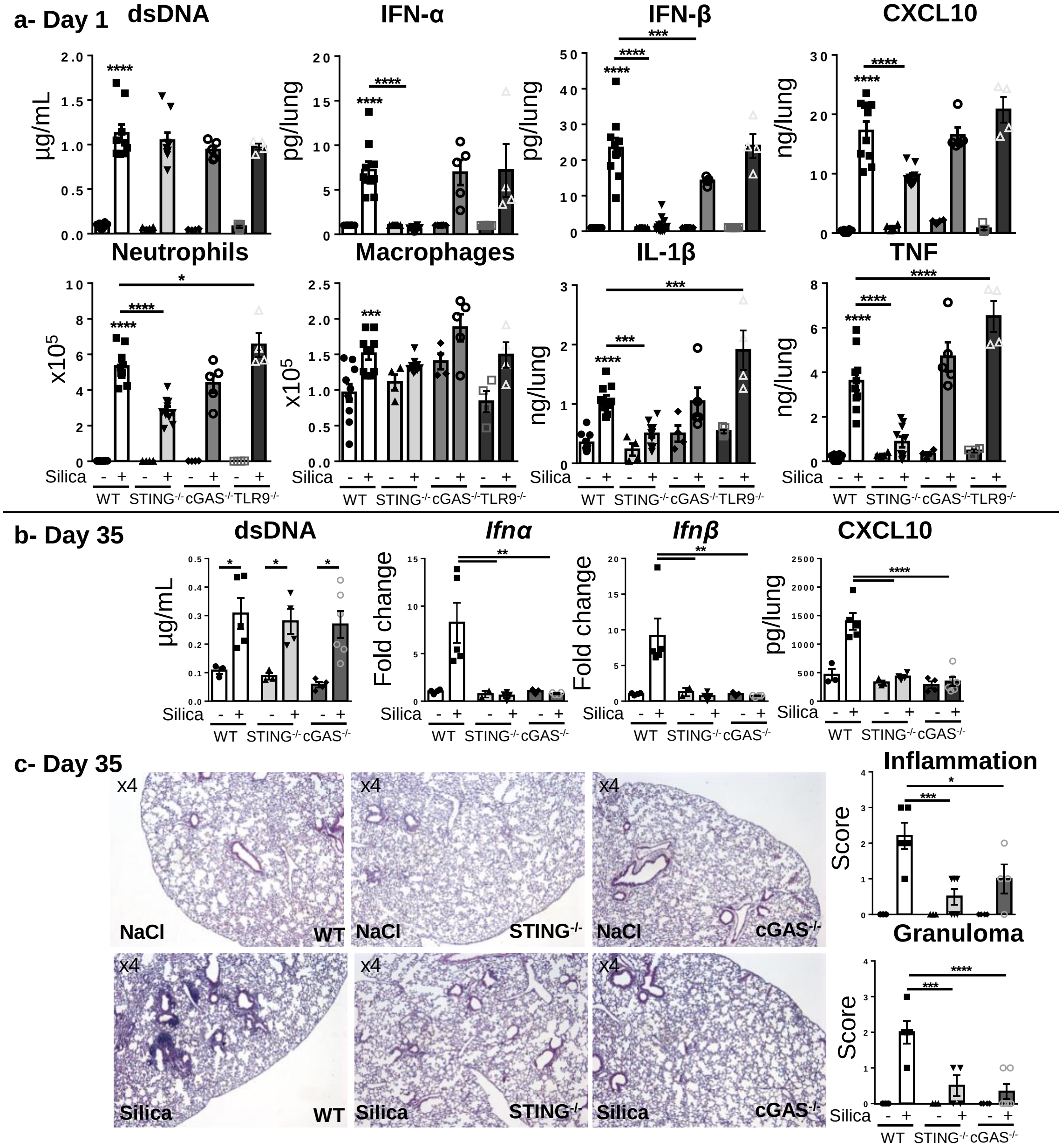

Supplementary Figure 3. STING is essential for silica-induced lung inflammation at an early time point, while cGAS is not.

(a) Silica microparticles (1 mg/mouse i.t.) or saline vehicle were administered to WT, STING$^{-/-}$, cGAS$^{-/-}$ and TLR9$^{-/-}$ mice and parameters analyzed on day 1 post-exposure.

Concentration of extracellular dsDNA in the bronchoalveolar lavage fluid (BALF) acellular fraction. Type I IFN levels quantified in lung homogenates by multiplex immunoassay and CXCL10, IL-1β and TNF by ELISA. Neutrophils and macrophages counts in the BALF.

(b-c) Silica microparticles (1 mg/mouse i.t.) or saline vehicle were administered to WT, STING$^{-/-}$, and cGAS$^{-/-}$ mice and parameters analyzed on day 35 post-exposure. (b) Concentration of extracellular dsDNA in the bronchoalveolar lavage fluid (BALF) acellular fraction. Lung levels of *Ifnα* and *Ifnβ* transcripts measured by RT-qPCR, and CXCL10 by ELISA. (c) Lung HE staining at day 35 post-silica exposure (4X magnification). The pathological scoring was established regarding the presence of necrotic cells, cell infiltration and granuloma.

p < 0.05, **p < 0.01, ***p < 0.001, ****p < 0.0001 (Kruskal-Wallis test followed by Dunn post-test). Data are presented as mean ± SEM and are representative of two independent experiments. (a: mice per group: n= 10 (WT NaCl), n= 10 (WT Silica), n= 4 (STING$^{-/-}$ NaCl), n= 9 (STING$^{-/-}$ Silica), n= 4 (cGAS$^{-/-}$ NaCl), n= 5 (cGAS$^{-/-}$ Silica), n= 5 (TLR9$^{-/-}$ NaCl), n= 4 (TLR9$^{-/-}$ Silica); b-c: mice per group: n= 3 (WT NaCl), n= 5 (WT Silica), n= 3 (STING$^{-/-}$ NaCl), n= 4 (STING$^{-/-}$ Silica), n= 4 (cGAS$^{-/-}$ NaCl), n= 6 (cGAS$^{-/-}$ Silica)). Source data are provided as a Source Data file.

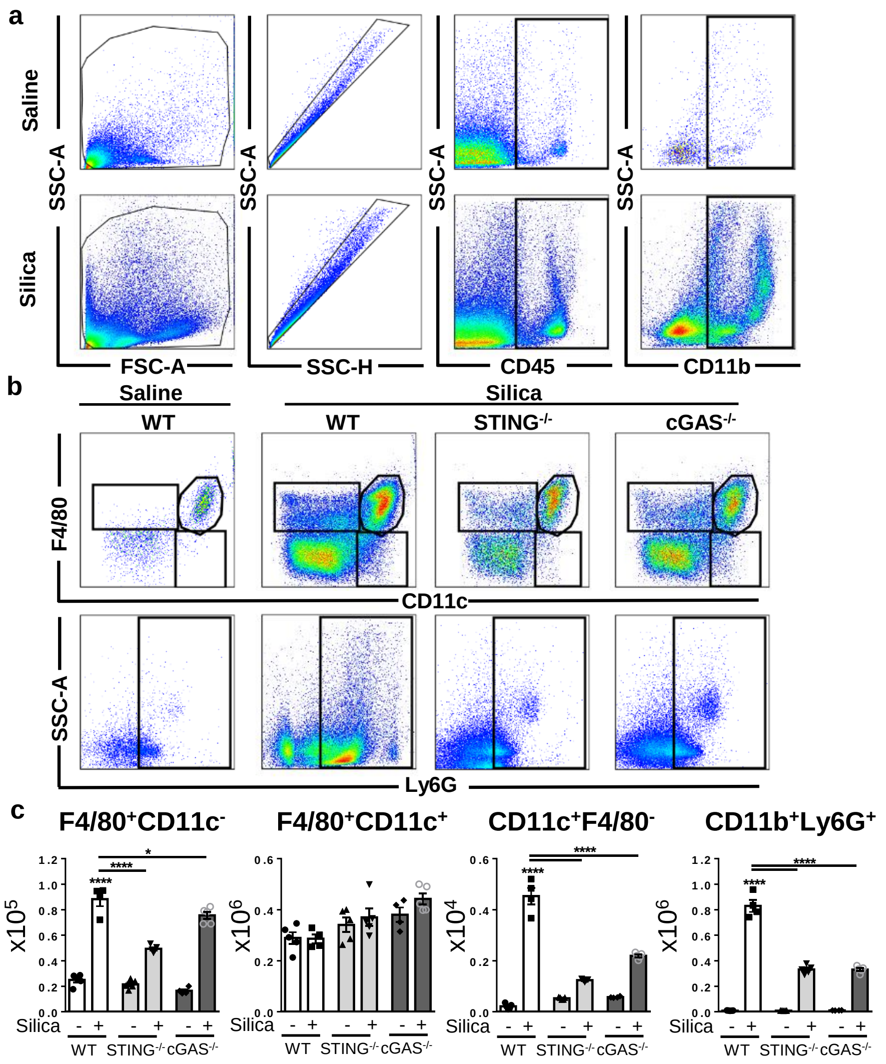

Supplementary Figure 4. cGAS/STING dependent recruitment of inflammatory cells in the airways after silica exposure.

Silica microparticles (1 mg/mouse i.t.) or saline vehicle were administered to WT, STING$^{-/-}$ and cGAS$^{-/-}$ mice and lung cell populations analysed on day 7 post-exposure.

(a-b) Gating strategy for flow cytometry analysis. Pre-gated singlet cells using SSC-A/SSC-H and CD45^{+}CD11b^{+} double positive cells are analysed for discriminating subcellular types: F4/80^{+}CD11c^{-} interstitial macrophages, F4/80^{+}CD11c^{+} alveolar macrophages, CD11c^{+}F4/80^{-} dendritic cells and Ly6G^{+}F4/80^{-} neutrophils.

(c) Quantification of interstitial macrophages, alveolar macrophages, dendritic cells and neutrophils per lung.

*p < 0.05, **p < 0.01, ***p < 0.001, ****p < 0.0001 (Kruskal-Wallis test followed by Dunn post-test). Data are presented as mean ± SEM and are representative of two independent experiments. Mice per group: n= 5 (WT NaCl), n= 4 (WT Silica), n= 5 (STING$^{-/-}$ NaCl), n= 5 (STING$^{-/-}$ Silica), n= 4 (cGAS$^{-/-}$ NaCl), n= 5 (cGAS$^{-/-}$ Silica). Each symbol represents an individual mouse. Source data are provided as a Source Data file.

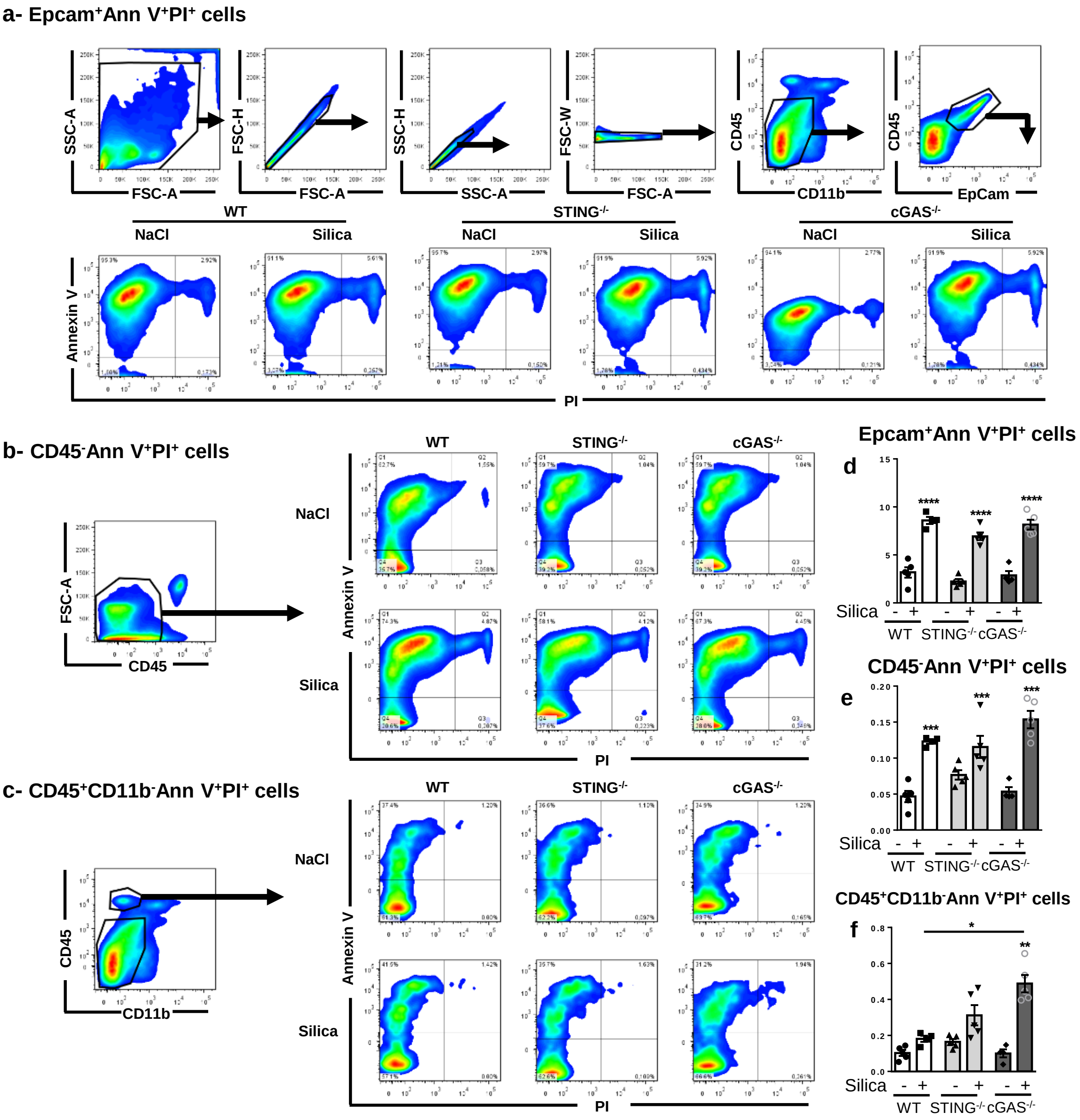

Supplementary Figure 5. cGAS/STING-independent epithelial cell death after silica exposure.

Silica microparticles (1 mg/mouse i.t.) or saline vehicle were administered to WT, STING$^{-/-}$ and cGAS$^{-/-}$ mice and parameters analyzed on day 7 post-exposure.

(a-c) Dot plots showing Annexin V/PI staining on CD45^{-}CD11b^{-}Epcam^{+} epithelial cells (a), CD45^{-} resident cells (b) and CD45^{+}CD11b^{-} lymphoid cells (c).

(d-f) Bargraphs of the Annexin/PI stained cells among epithelial cells (d), CD45^{-} cells (e) and CD45^{+}CD11b^{-} cells (f), expressed as absolute cell number per lung.

*p < 0.05, **p < 0.01, ***p < 0.001, ****p < 0.0001 (one-way ANOVA with Tukey's post hoc test). Data are presented as mean ± SEM and are representative of two independent experiments (mice per group: n= 5 (WT NaCl), n= 4 (WT Silica), n= 5 (STING$^{-/-}$ NaCl), n= 5 (STING$^{-/-}$ Silica), n= 4 (cGAS$^{-/-}$ NaCl), n= 5 (cGAS$^{-/-}$ Silica)). Each symbol represents an individual mouse. Source data are provided as a Source Data file.

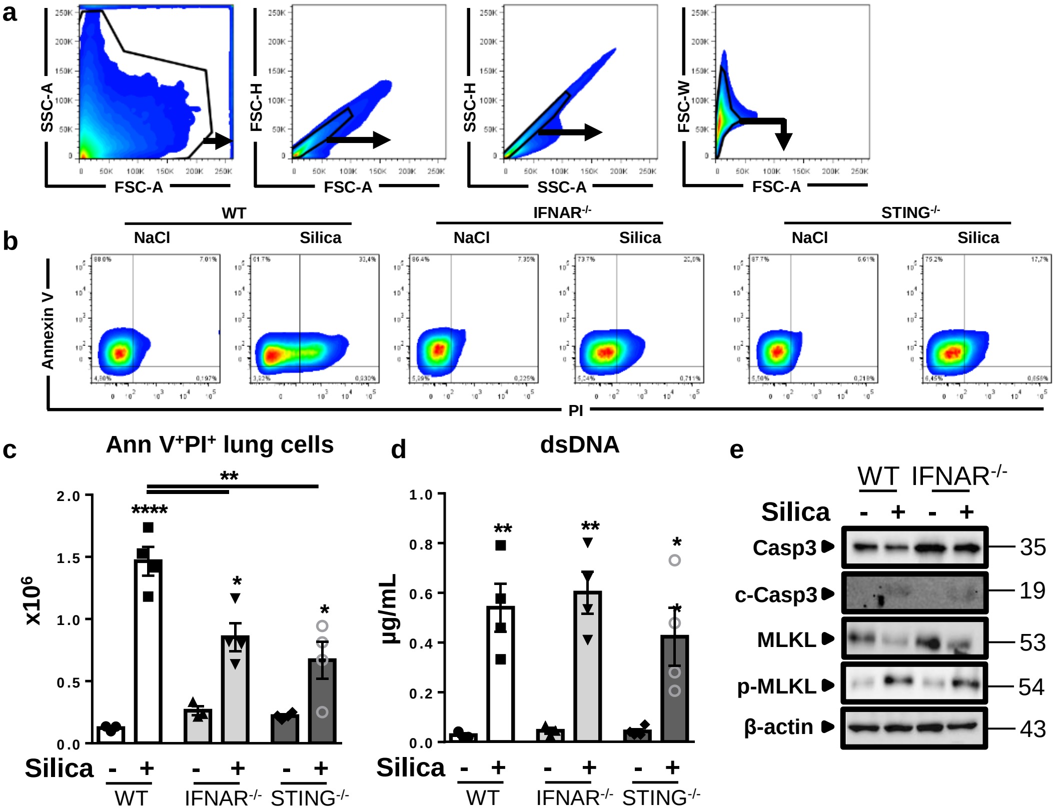

Supplementary Figure 6. Partial contribution of Type I IFN signaling in early silica-induced lung cell death.

Silica microparticles (1 mg/mouse i.t.) or saline vehicle were administered to WT, IFNAR$^{-/-}$ and STING$^{-/-}$ mice and parameters analyzed on day 1 post-exposure.

(a) Gating strategy showing the exclusion of doublet cells using FSC-H/FSC-A, SSC-H/SSC-A followed by FSC-W/FSC-A.

(b) Dot plots showing Ann V$^+$/PI$^+$ dead lung cells.

(c) Absolute numbers of Ann V$^+$/PI$^+$ cells per lung.

(d) Concentration of extracellular dsDNA in the BALF.

(e) Immunoblots of caspase 3, cleaved caspase 3, phospho-MLKL, MLKL and β-actin as a reference.

*$p < 0.05$, **$p < 0.01$, ***$p < 0.001$, ****$p < 0.0001$ (one-way ANOVA with Tukey's post hoc test). Data are presented as mean ± SEM (mice per group: n= 3 (WT NaCl), n= 4 (WT Silica), n= 3 (IFNAR$^{-/-}$ NaCl), n= 4 (IFNAR$^{-/-}$ Silica), n= 4 (STING$^{-/-}$ NaCl), n= 4 (STING$^{-/-}$ Silica)). Immunoblots representative of n= 2 samples. Each symbol represents an individual mouse. Source data are provided as a Source Data file.

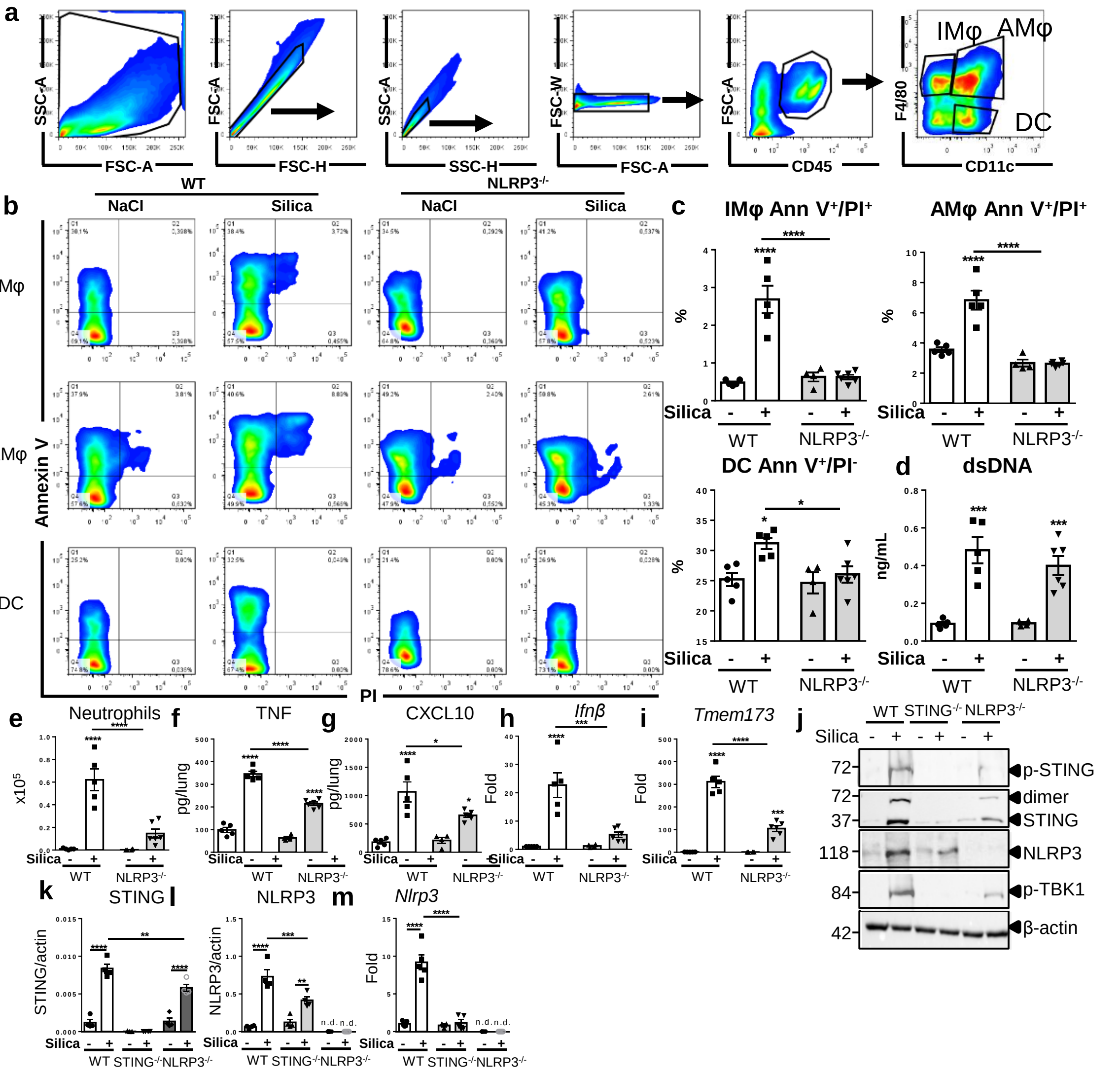

Supplementary Figure 7. Interplay of STING and NLRP3 to promote silica-induced lung inflammation.

(a-f) Silica microparticles (1 mg/mouse i.t.) or saline vehicle were administered to WT and NLRP3$^{-/-}$ mice and parameters analyzed on day 7 post-exposure. (a) Gating strategy for flow cytometry analysis. Pre-gated singlet cells using FSC-A/FSC-H followed by SSC-A/SSC-H and FSC-W/FSC-A. CD45 positive cells were gated and stained followed by F4/80 and CD11c for discriminating subcellular types: F4/80^{+}CD11c^{-} interstitial macrophages, F4/80^{+}CD11c^{+} alveolar macrophages and CD11c^{+}F4/80^{-} dendritic cells.

(b) Dot plots showing Ann V^{+}/PI^{+} interstitial and alveolar macrophages and Ann V^{+}/PI^{-} DC. (c) Proportion of dead cells (Ann V^{+}/PI^{+}) among interstitial and alveolar macrophages and proportion of early apoptotic cells (Ann V^{+}/PI^{-}) among DC (on 200 000 events).

(d) Concentration of extracellular dsDNA in the BALF acellular fraction.

(e) Neutrophils count in the BALF. Lung (f) TNF and (g) CXCL10 levels measured by ELISA, and (h) *Ifnβ* and (i) *Tmem173* transcripts measured by real-time PCR.

(j-m) Silica microparticles (1 mg/mouse i.t.) or saline vehicle were administered to WT, STING$^{-/-}$ and NLRP3$^{-/-}$ mice and lung parameters analyzed on day 7 post-exposure.

(j) Immunoblots of phospho-STING, STING, phospho-TBK1, NLRP3 and β-actin as a reference. Relative quantification of (k) STING and (l) NLRP3 from immunoblots, as compared to β-actin. (m) Nlrp3 transcripts measured by real-time PCR.

*p < 0.05, **p < 0.01, ***p < 0.001, ****p < 0.0001 (one-way ANOVA with Tukey's post hoc test). Data are presented as mean ± SEM (a-i: mice per group: n= 5 (WT NaCl), n= 5 (WT Silica), n= 4 (NLRP3$^{-/-}$ NaCl), n= 6 (NLRP3$^{-/-}$ Silica); k-m: mice per group: n= 4 (WT NaCl), n= 5 (WT Silica), n= 4 (STING$^{-/-}$ NaCl), n= 4 (STING$^{-/-}$ Silica), n= 4 (NLRP3$^{-/-}$ NaCl), n= 5 (NLRP3$^{-/-}$ Silica)). Immunoblots are representative of n= 4 samples (j), quantified in bargraphs (k,l). Each symbol represents an individual mouse. n.d. not detected. Source data are provided as a Source Data file.

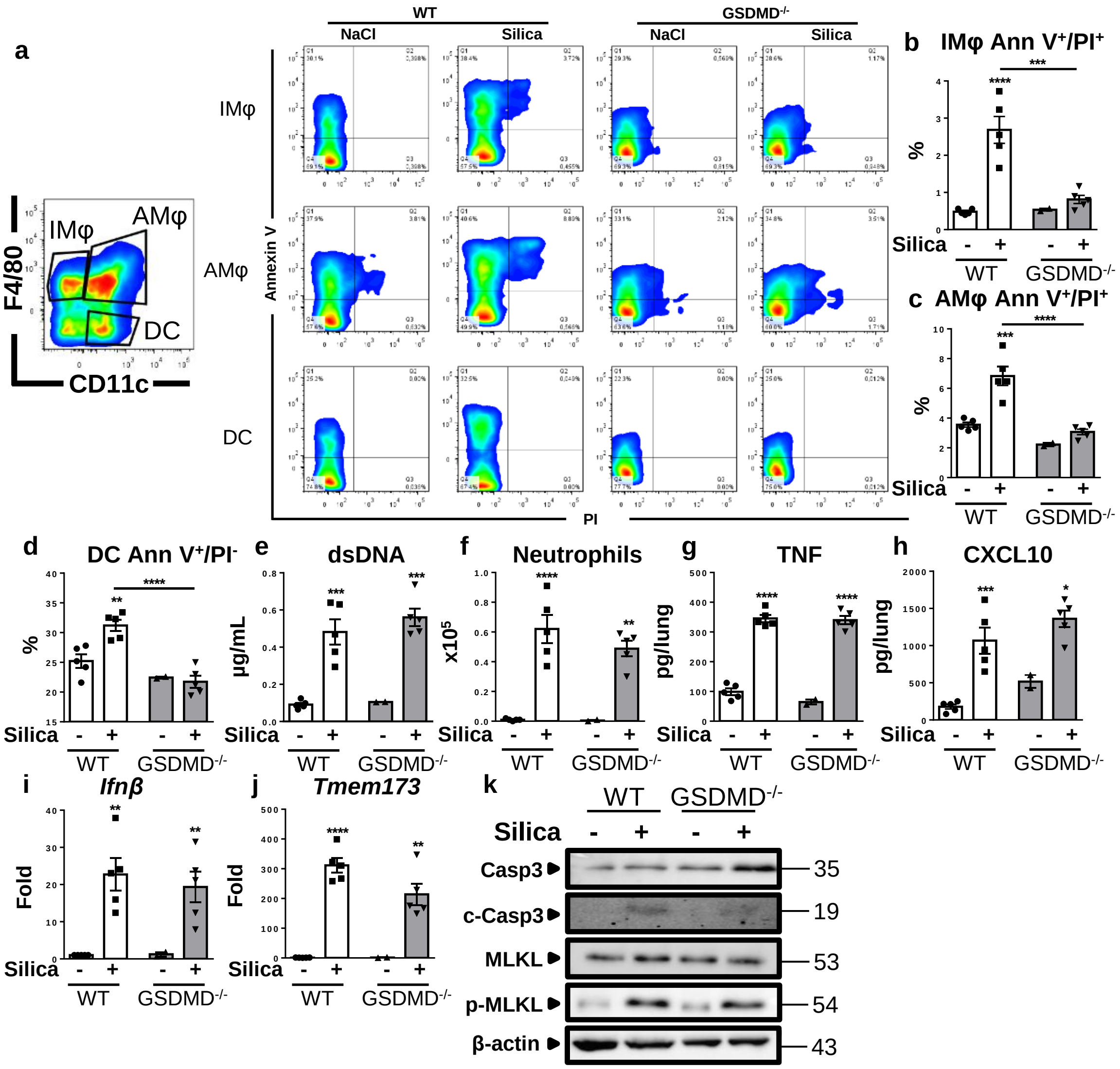

Supplementary Figure 8. Silica induced lung inflammation in the absence of Gasdermin D.

Silica microparticles (1 mg/mouse i.t.) or saline vehicle were administered to WT and GSDMD$^{-/-}$ mice and parameters analyzed on day 7 post-exposure.

(a) Dot plots showing Annexin V/PI staining on interstitial macrophages (IMφ), alveolar macrophages (AMφ) and dendritic cells (DC).

(b-d) Proportion of Ann V^{+}/PI^{+} IMφ, AMφ and DC.

(e) Concentration of extracellular dsDNA in the BALF.

(f) Neutrophils count in the BALF. Lung (g) TNF and (h) CXCL10 levels measured by ELISA, and (i) *Ifnβ* and (j) *Tmem173* transcripts measured by real-time PCR.

(k) Immunoblots of caspase 3, cleaved caspase 3, phospho-MLKL, MLKL and β-actin as a reference.

*p < 0.05, **p < 0.01, ***p < 0.001, ****p < 0.0001 (one-way ANOVA with Tukey's post hoc test). Data are presented as mean ± SEM (mice per group: n= 5 (WT NaCl), n= 5 (WT Silica), n= 2 (GSDMD$^{-/-}$ NaCl), n= 5 (GSDMD$^{-/-}$ Silica)). Immunoblots are representative of n= 2 samples. Each symbol represents an individual mouse. Source data are provided as a Source Data file.

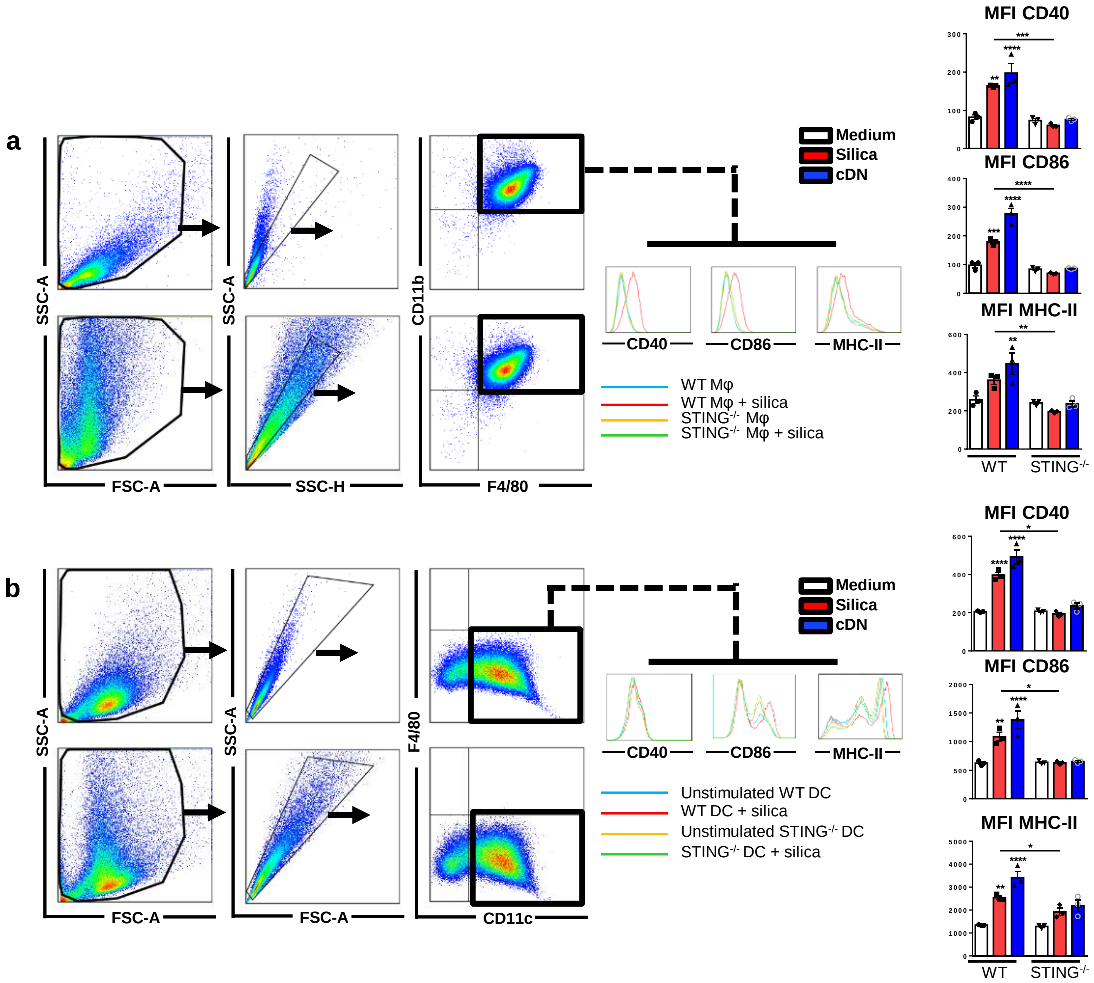

Supplementary Figure 9. STING is crucial for APCs activation after silica in vitro exposure.

Bone marrow-derived macrophages (a) or DC (b) from WT and STING$^{-/-}$ mice were unstimulated, stimulated with silica (250 µg/mL), or transfected with c-di-AMP (6 µg/mL; cDN) for 18 h.

(a) Singlet CD11b^{+}F4/80^{+}CD11c^{-} WT or STING$^{-/-}$ macrophages were analysed by flow cytometry for CD40, CD86 and IA/IE expression. Representative histograms and quantification of the mean fluorescence intensity (MFI) for CD40, CD86 and IA/IE. (b) Singlet CD11b^{+}F4/80^{-}CD11c^{+} WT or STING$^{-/-}$ DC analysed as in (a).

*$p < 0.05$, **$p < 0.01$, ***$p < 0.001$, ****$p < 0.0001$ (Mann-Whitney U analysis). Data are presented as mean ± SEM and are representative of two independent experiments with similar results (a-b) or with n= 3 independent cultures (a-b). Each symbol represents an individual technical replicate. Source data are provided as a Source Data file.

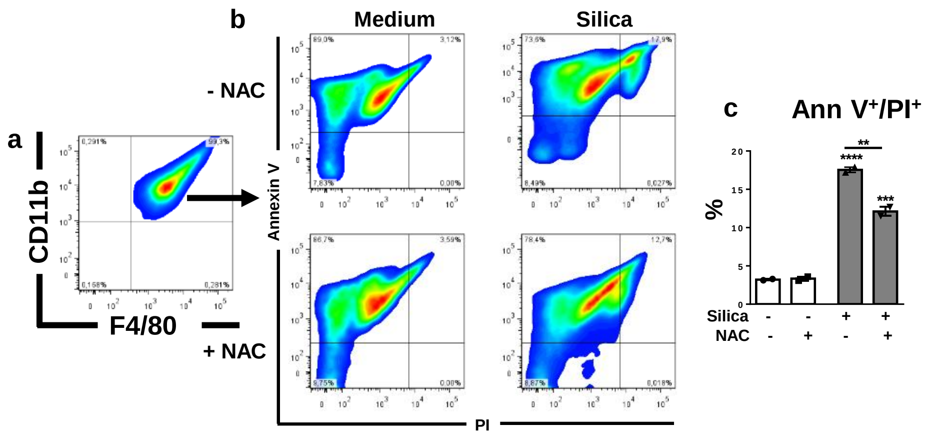

Supplementary Figure 10. ROS scavenger inhibits macrophages death after in vitro silica exposure.

Bone marrow-derived macrophages from WT mice were treated with N-acetylcysteine (NAC) during 1h prior to silica exposure (250µg/mL) for 18 hr. The purity of the macrophage population was verified by flow cytometry using CD11b, CD11c and F4/80 staining: 99.7±0.5% $CD11b^+F4/80^+CD11c^-$ cells (a).

(b-c) Dot plots showing Annexin V/PI staining and (c) proportion of Ann V^+/PI^+ macrophages in the different groups.

**p < 0.01, ***p < 0.001, ****p < 0.0001 (Mann-Whitney U analysis). Data are presented as mean ± SD of n= 2 independent cultures. Source data are provided as a Source Data file.

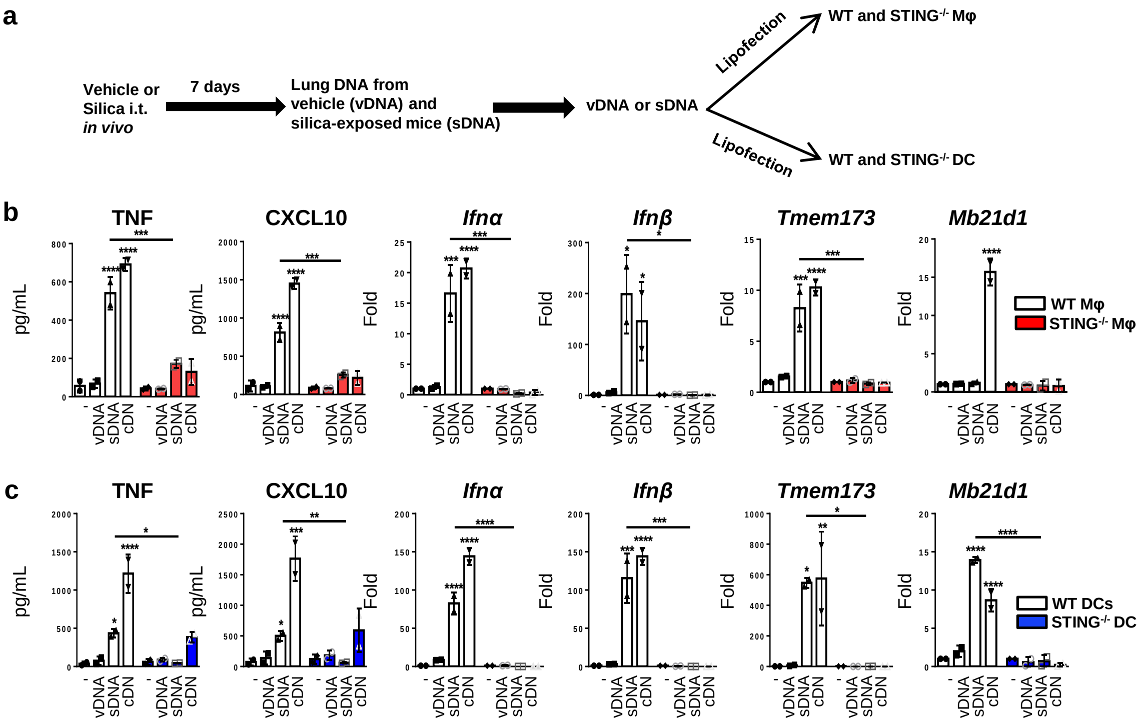

Supplementary Figure 11. Silica-induced self-dsDNA acts as a DAMP activating APC type I IFN response.

(a) Self-dsDNA purified from the lung of WT mice 7 days after i.t. exposure to silica microparticles (1 mg/mouse; sDNA) or saline vehicle (vDNA) was transfected for 6 hr into WT and STING$^{-/-}$ BMDM (b) and BMDC (c), using transfected c-di-AMP (6 µg/mL; cDN) as a positive control.

(b-c) Concentration of TNFα and CXCL10 determined by ELISA in BMDM (b) and BMDC (c) culture supernatant. *Ifnα*, *Ifnβ*, *Tmem173* and *Mb21d1* transcripts quantified by real-time PCR on cell fractions.

*$p < 0.05$, **$p < 0.01$, ***$p < 0.001$, ****$p < 0.0001$ (Mann-Whitney U analysis). Data are presented as mean ± SD and are representative of two independent experiments with n= 2 independent cultures. Each symbol represents an individual technical replicate. Source data are provided as a Source Data file.

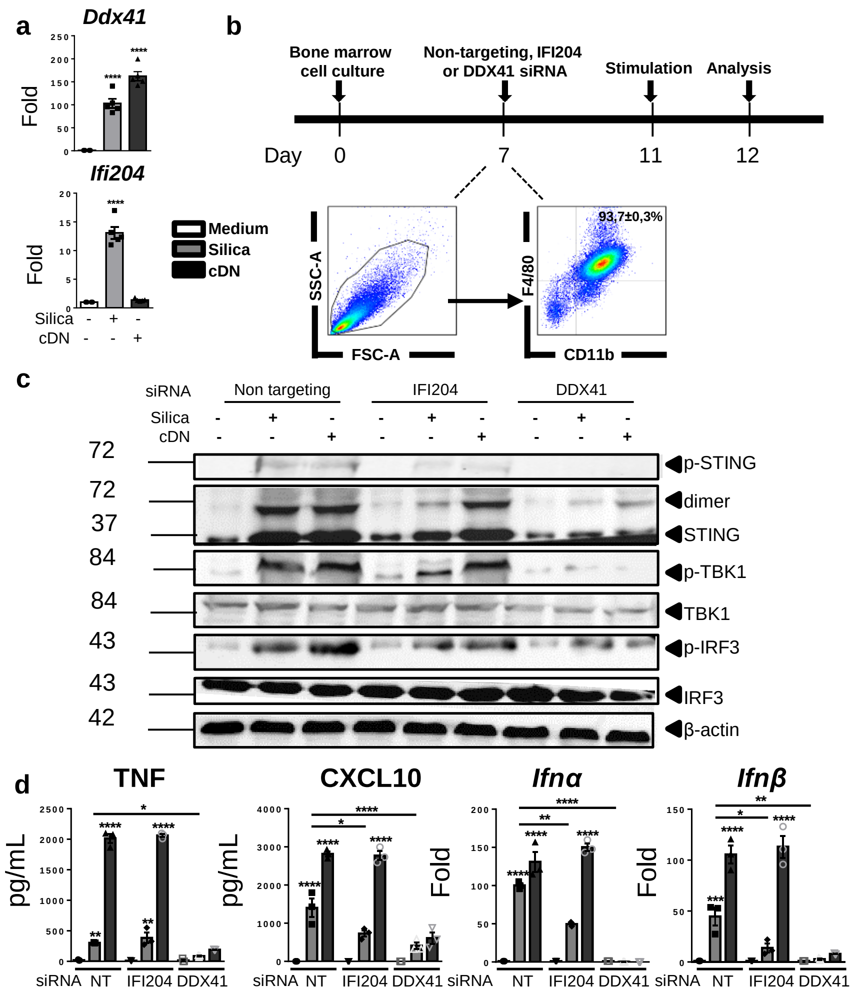

Supplementary Figure 12. DDX41 and IFI204 activate STING signaling pathway leading to type I IFNs response in silica-stimulated macrophages.

(a) Transcripts for *Ifi204* and *Ddx41* measured by real-time PCR in WT bone marrow-derived macrophages unstimulated, stimulated with silica (250 µg/mL) or transfected with c-di-AMP (6 µg/mL; cDN) for 18 hr.

(b) WT bone marrow cells were treated with silencing RNA targeting IFI204, DDX41 and non-targeting RNA after harvesting on day 7 of culture in DMEM medium supplemented with 10% FCS, 100 U/mL penicillin and 100 µg/mL streptomycin, 2 mM glutamine, plus 20% horse serum and 30% (v/v) L929 conditioned medium as a source of M-CSF. On day 11, after further 4 days in culture, BMDM were stimulated as in (a). The purity of the BMDM culture was verified by flow cytometry on day 7 using CD11b, CD11c and F4/80 staining: 93.7±0.3% $CD11b^+F4/80^+CD11c^-$ cells.

(c) Immunoblots of STING/IRF3 axis including phospho-STING, STING, phospho-TBK1, TBK1, phospho-IRF3, IRF3, with β-actin as a control in BMDM pre-treated with siRNA targeting IFI204, DDX41 as in (b).

(d) Concentrations of TNFα and CXCL10 quantified by ELISA in culture supernatant. *Ifnα* and *Ifnβ* transcripts measured on cell fraction by real-time PCR.

*p < 0.05, **p < 0.01, ***p < 0.001, ****p < 0.0001 (Mann-Whitney U analysis). Data are presented as mean ± SD with n= 2 independent cultures. Immunoblots are representative of n= 3 independent cultures (c). Each symbol represents an individual technical replicate. Source data are provided as a Source Data file.

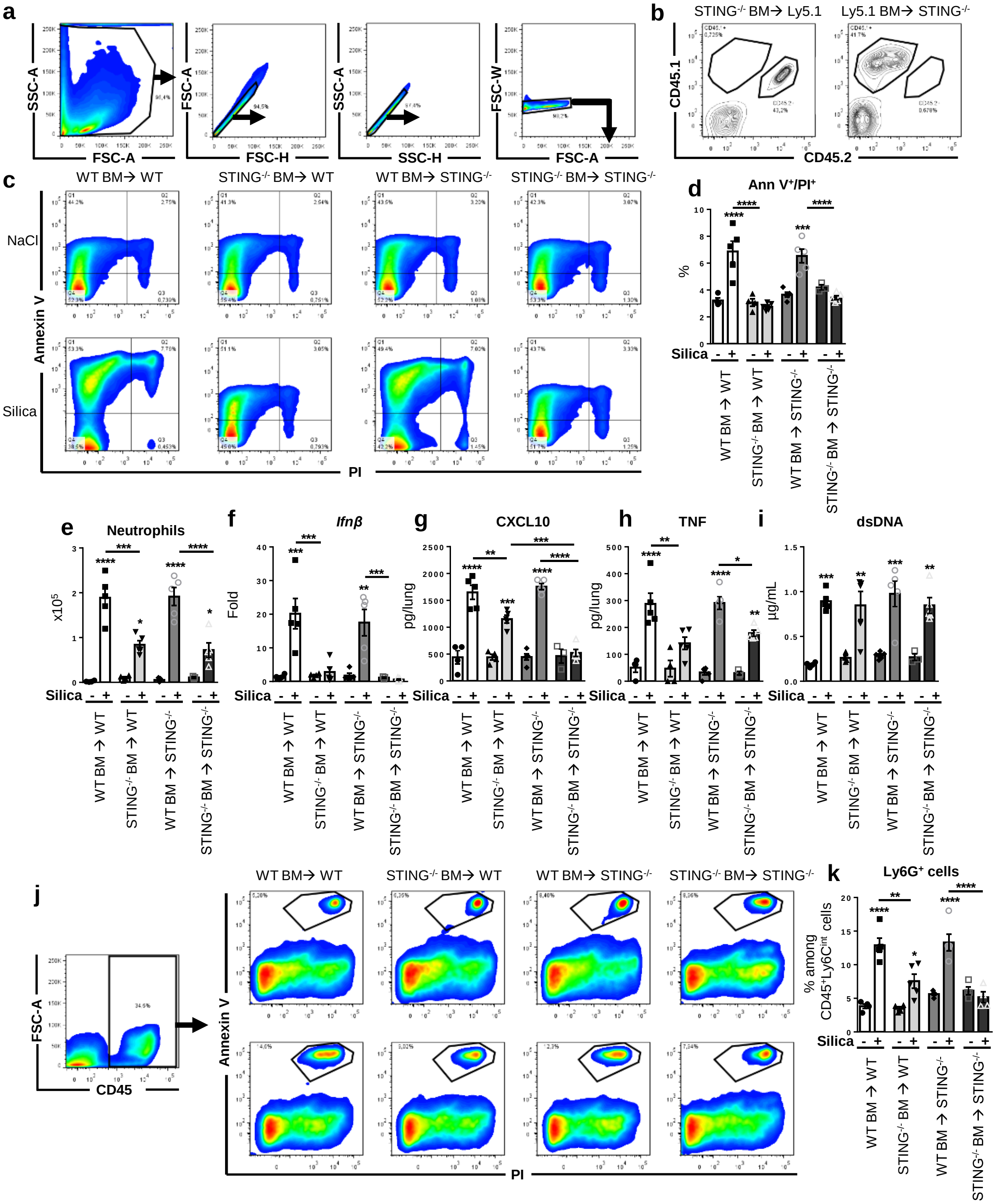
a
SSC-A
FSC-A
FSC-H
SSC-H
FSC-W
b
STING-/- BM→ Ly5.1
Ly5.1 BM→ STING-/-
CD45.1
CD45.2
c
WT BM→ WT
STING-/- BM→ WT
WT BM→ STING-/-
STING-/- BM→ STING-/-
NaCl
Silica
Annexin V
PI
d
Ann V+/PI+
%
Silica
WT BM → WT
STING-/- BM → WT
WT BM → STING-/-
STING-/- BM → STING-/-
e
Neutrophils
x10^5
f
Ifnβ
Fold
g
CXCL10
pg/lung
h
TNF
pg/lung
i
dsDNA
µg/mL
j
FSC-A
CD45
Annexin V
PI
k
Ly6G+ cells
% among CD45+Ly6Cint cells

Supplementary Figure 13. Hematopoietic STING drives silica-induced lung inflammation.

$STING^{-/-}$ mice and WT mice were lethally irradiated and reconstituted with bone marrow from either WT mice or $STING^{-/-}$ mice before silica microparticles (1 mg/mouse i.t.) or saline administration. Parameters were analyzed on day 7 post-exposure.

(a-b) Control of hematopoietic reconstitution. (a) Gating strategy for flow cytometry analysis. Pre-gated singlet cells using FSC-A/FSC-H followed by SSC-A/SSC-H and FSC-W/FSC-A. (b) Whole blood staining using CD45.1 for WT Ly5.1 cells and CD45.2 for $STING^{-/-}$ cells.

(c) Dot plots showing Annexin V/PI staining of lung cells and (d) proportion of dead lung cells (Ann V^{+}/PI^{+}) in the different groups.

(e) Neutrophils count in the BALF. (f) Lung *Ifnβ* transcripts quantified by real-time PCR and (g) CXCL10 and (h) TNF levels measured by ELISA.

(i) Concentration of extracellular dsDNA in the BALF acellular fraction.

Dot plots (j) and (k) proportion of $CD45^{+}Ly6G^{high}Ly6C^{int}$ granulocytes in the lungs (on 200 000 events).

*$p < 0.05$, **$p < 0.01$, ***$p < 0.001$, ****$p < 0.0001$ (one-way ANOVA with Tukey's post hoc test). Data are presented as mean ± SEM (mice per group: n= 4 (WT BM→ WT NaCl), n= 5 (WT BM→ WT Silica), n= 4 ($STING^{-/-}$ BM→WT NaCl), n= 5 ($STING^{-/-}$ BM→WT Silica), n= 3 (WT BM→$STING^{-/-}$ NaCl), n= 4 (WT BM→$STING^{-/-}$ Silica), n= 3 ($STING^{-/-}$ BM→$STING^{-/-}$ NaCl), n= 5 ($STING^{-/-}$ BM→$STING^{-/-}$ Silica)). Each symbol represents an individual mouse. Source data are provided as a Source Data file.

Supplementary Figure 14. Uncropped immunoblots of the different figures.

Figure 1

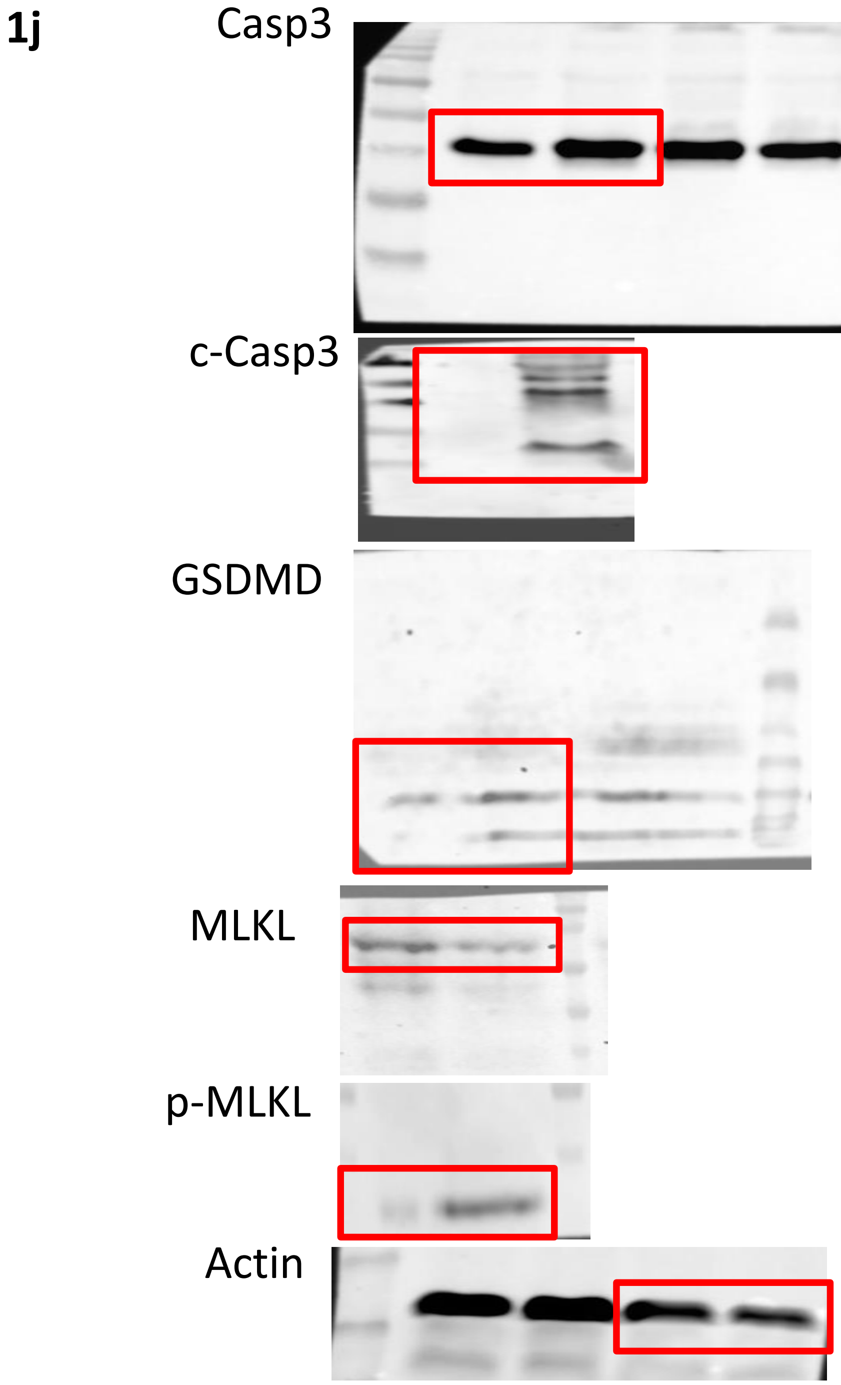

Figure 1

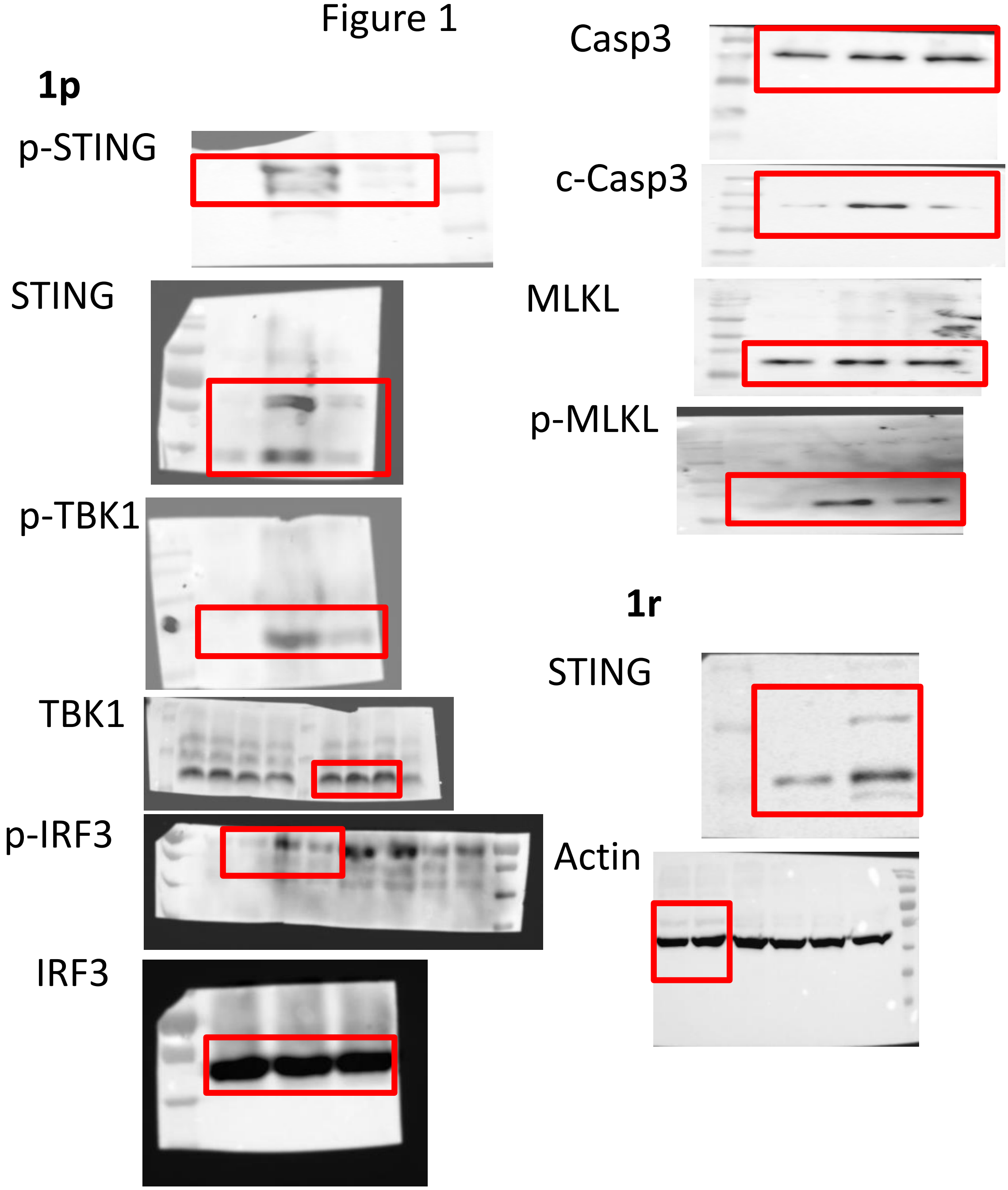

1p
p-STING
STING
p-TBK1
TBK1
p-IRF3
IRF3
Casp3
c-Casp3
MLKL
p-MLKL
1r
STING
Actin

Figure 2

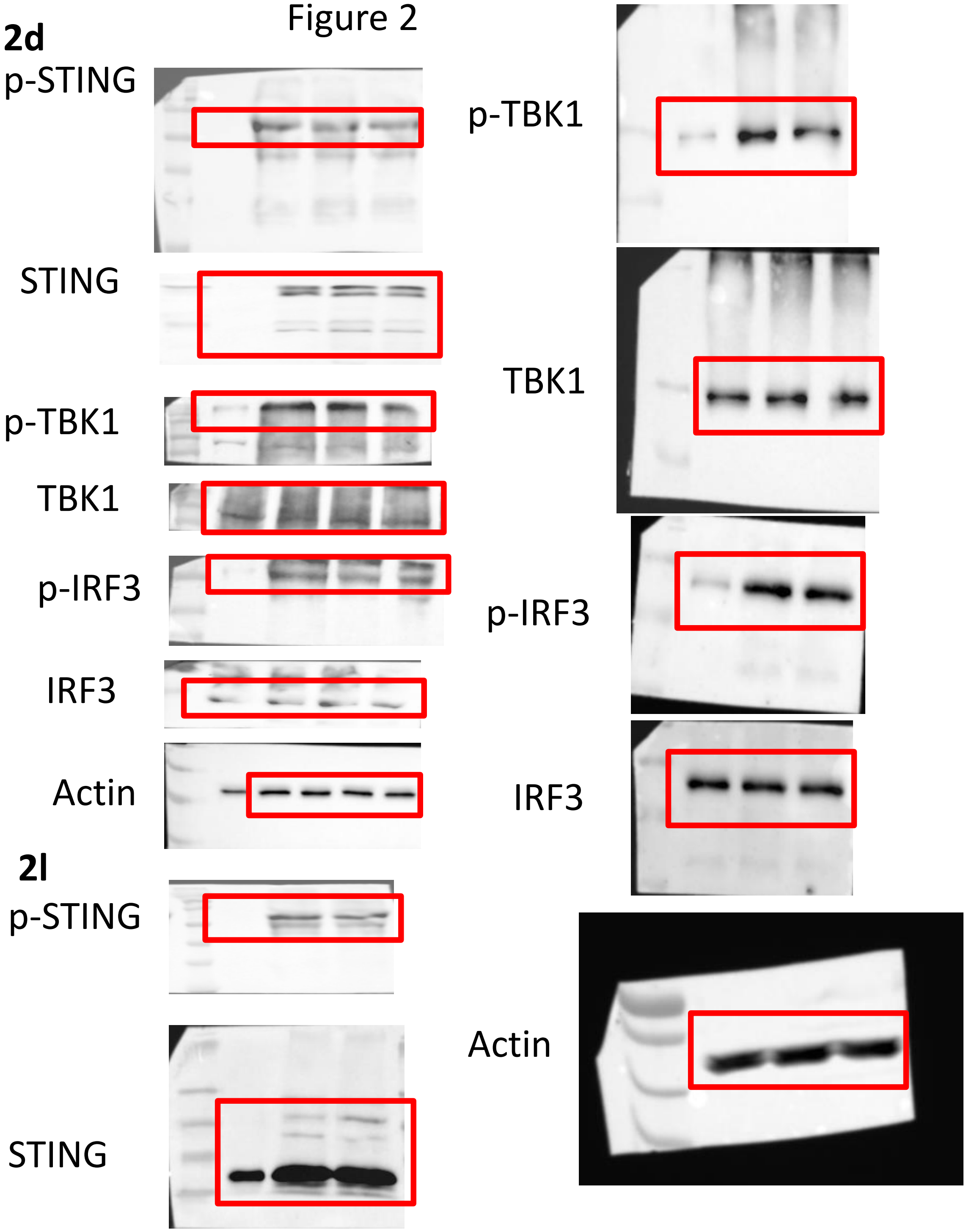
2d
p-STING
STING
p-TBK1
TBK1
p-IRF3
IRF3
Actin
2l
p-STING
STING
p-TBK1
TBK1
p-IRF3
IRF3
Actin

Figure 3

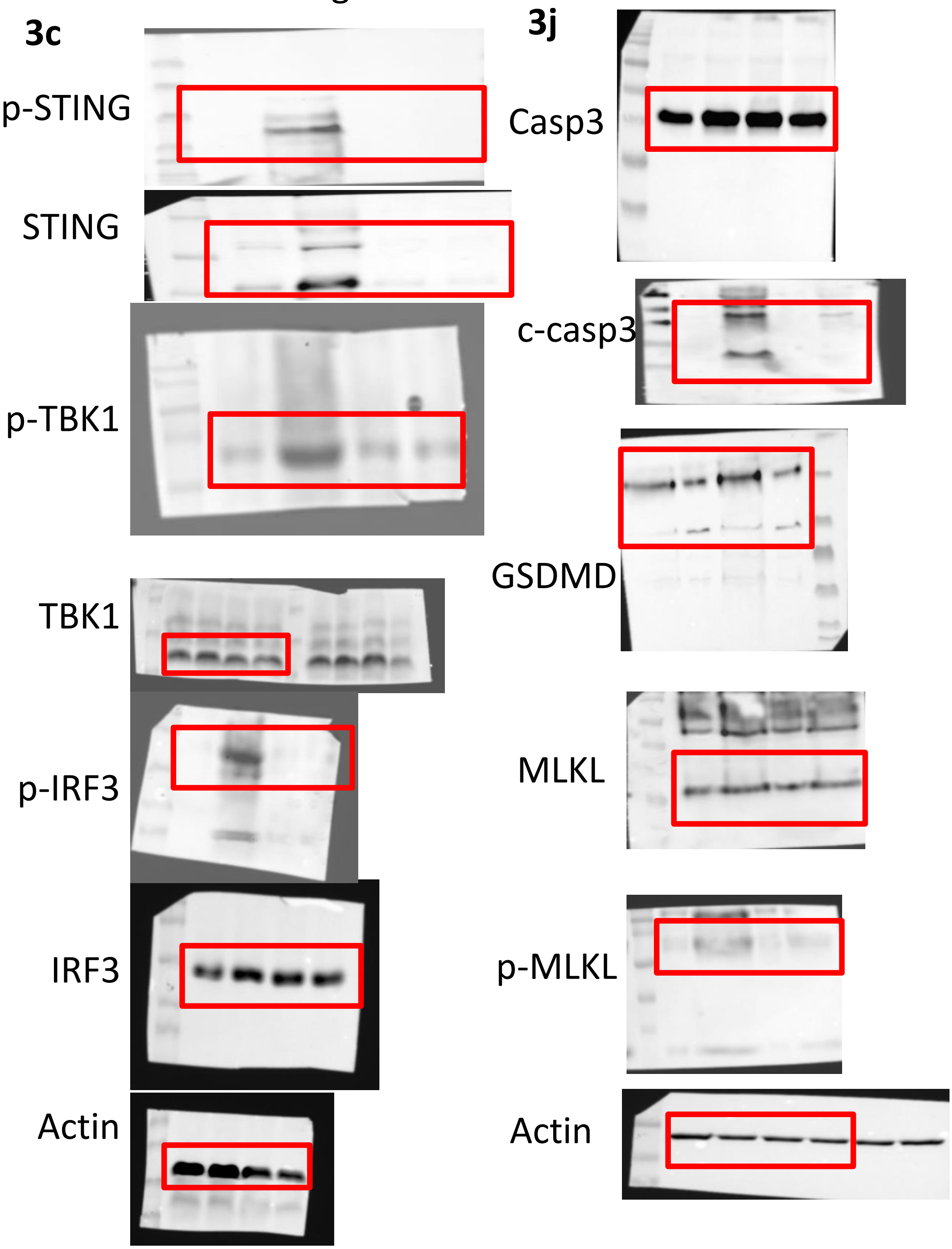
3c
p-STING
STING
p-TBK1
TBK1
p-IRF3
IRF3
Actin
3j
Casp3
c-casp3
GSDMD
MLKL
p-MLKL
Actin

Figure 4

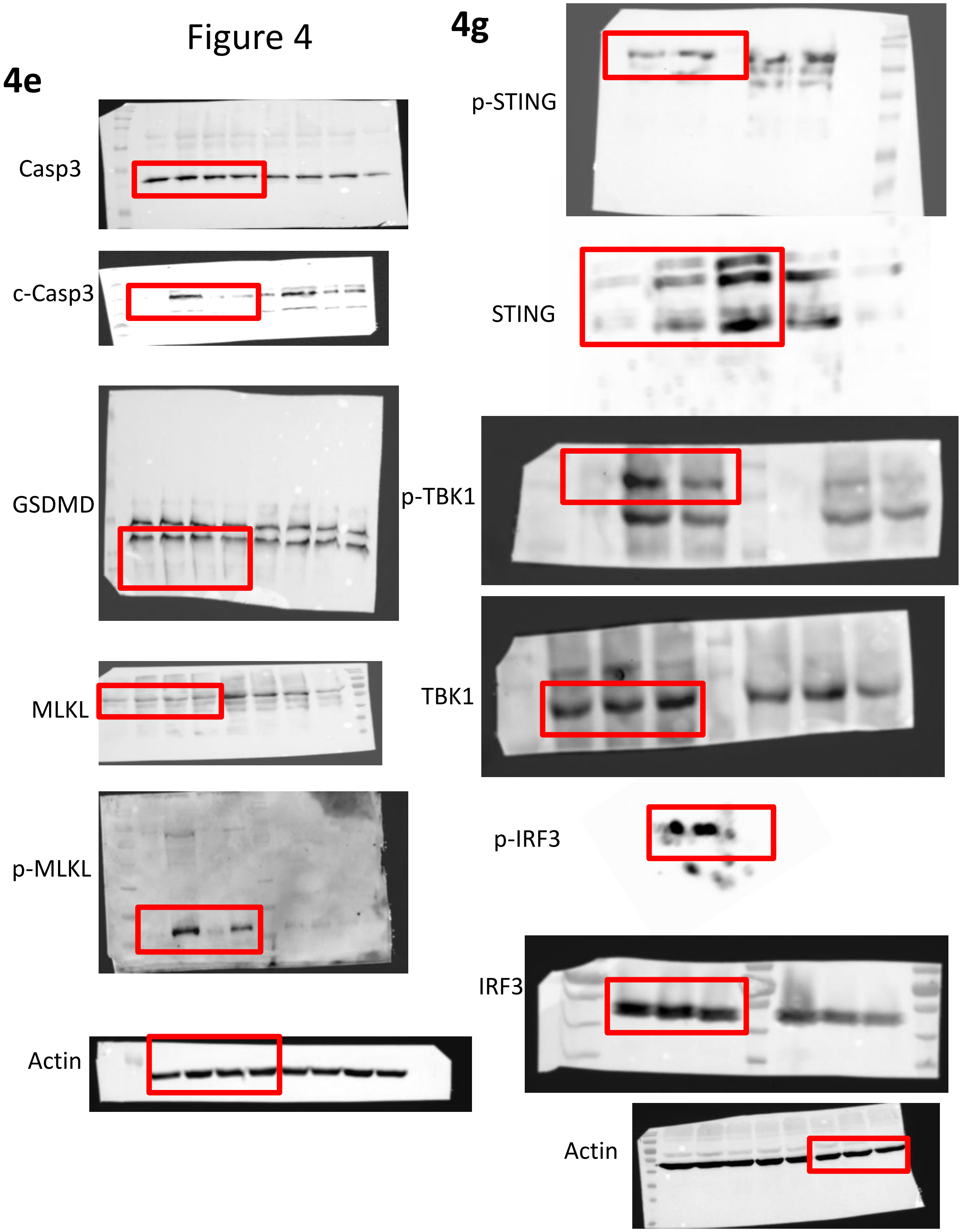
4e
Casp3
c-Casp3
GSDMD
MLKL
p-MLKL
Actin
4g
p-STING
STING
p-TBK1
TBK1
p-IRF3
IRF3
Actin

Figure 5

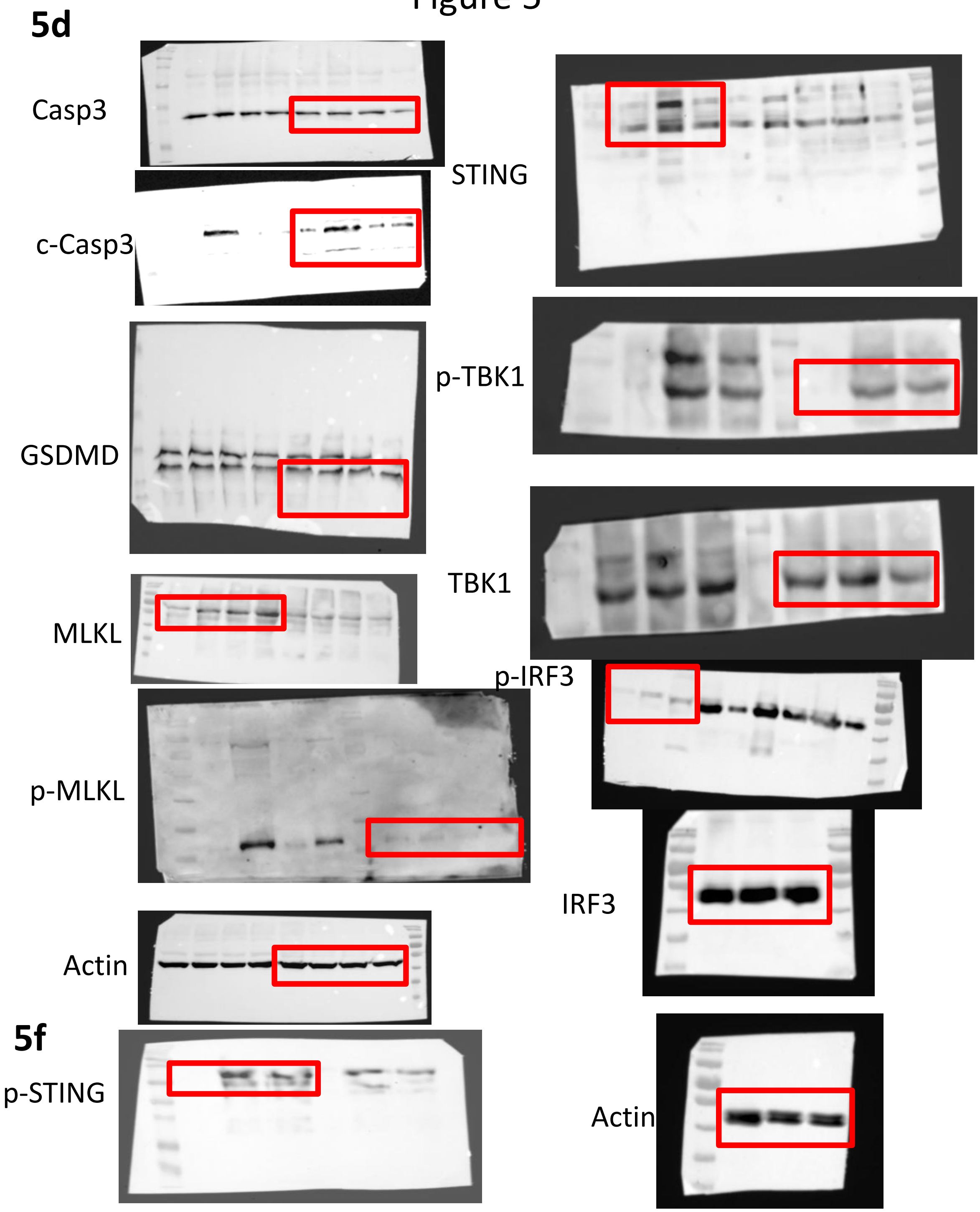
5d
Casp3
c-Casp3
GSDMD
MLKL
p-MLKL
Actin
STING
p-TBK1
TBK1
p-IRF3
IRF3
Actin
5f
p-STING

6g

Figure 6

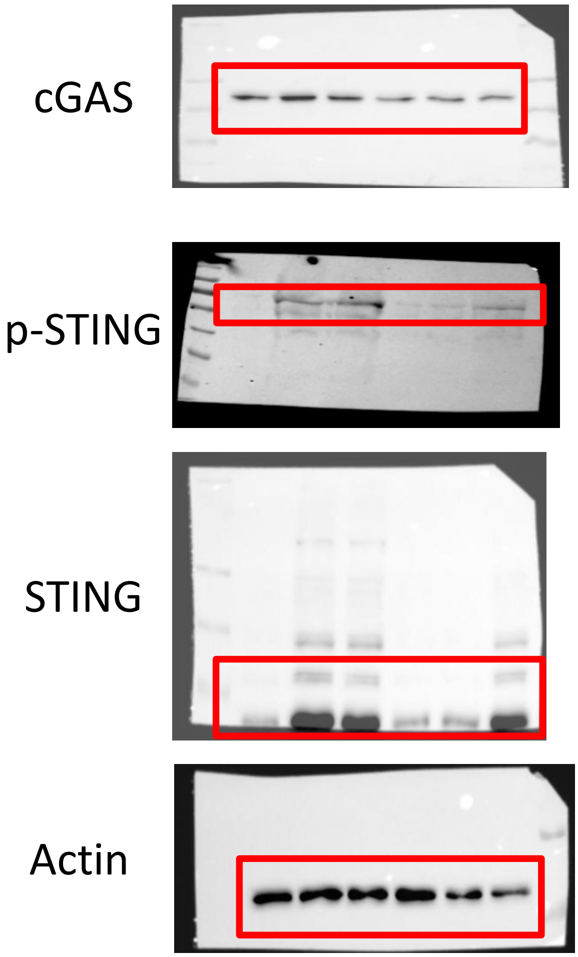

S2f Supplementary Figure 2

p-STING

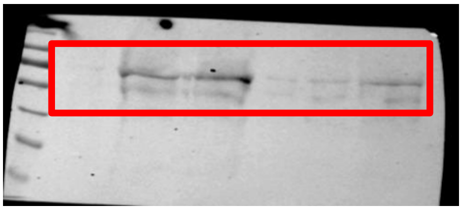

STING

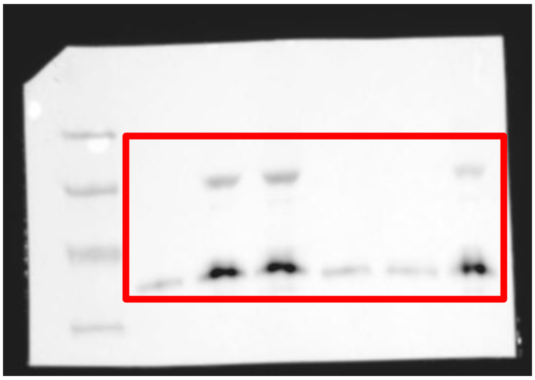

Actin

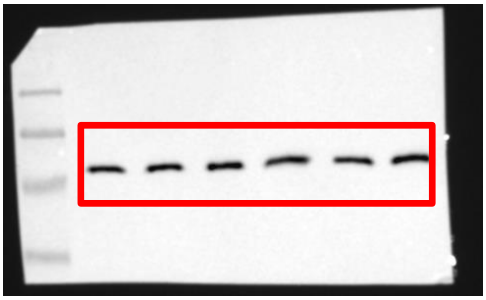

Supplementary Figure 6

S6e

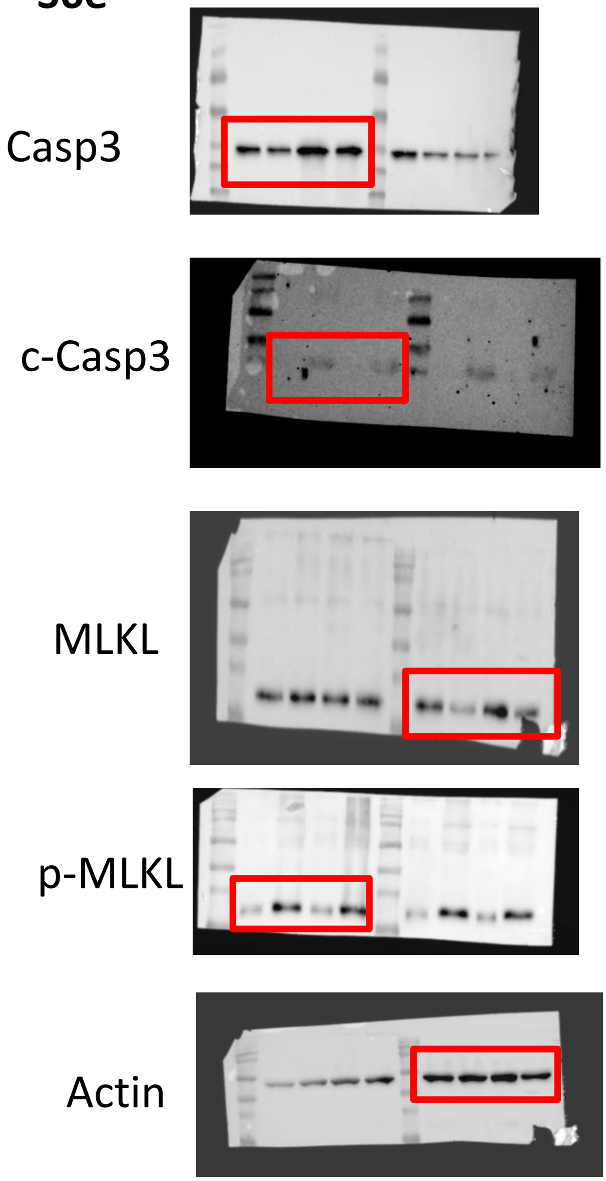

Supplementary Figure 7

S7j

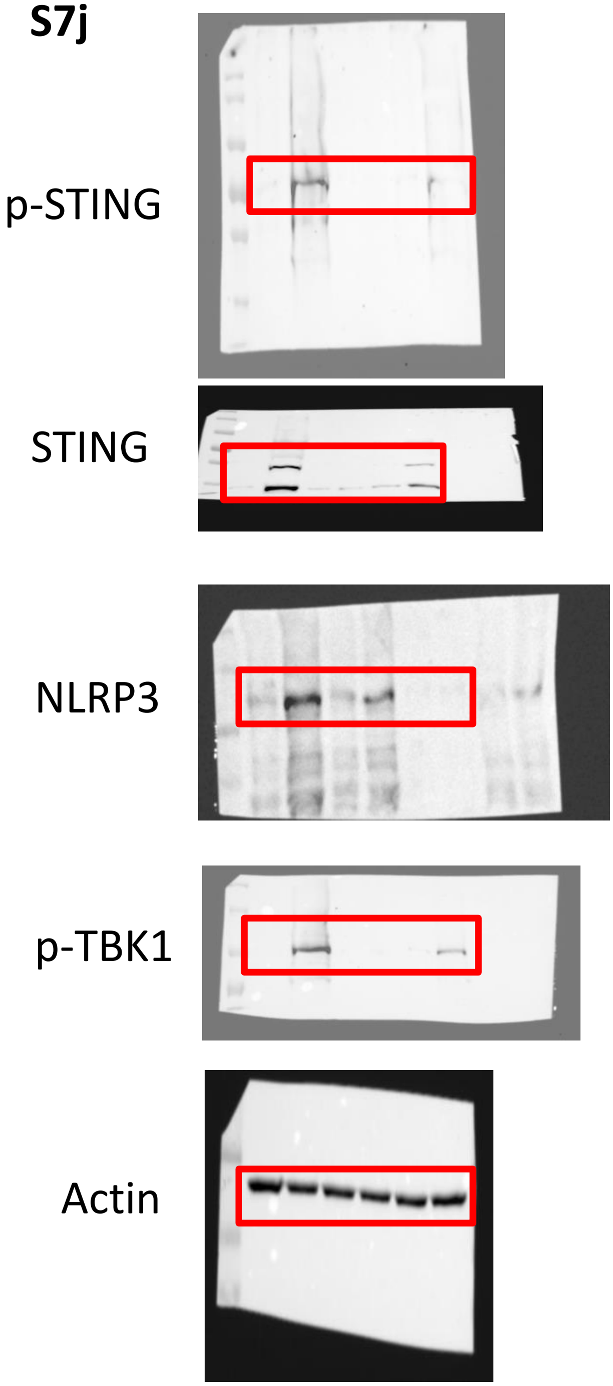

Supplementary Figure 8

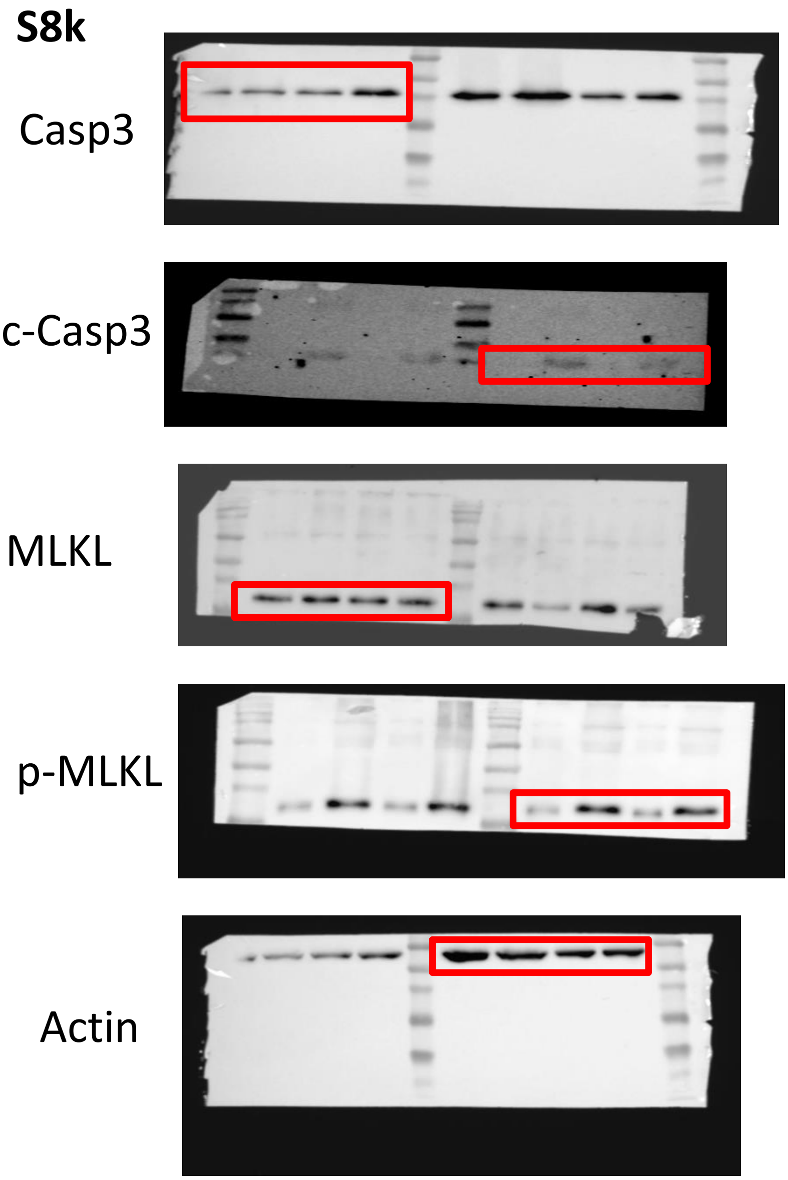

Supplementary Figure 12

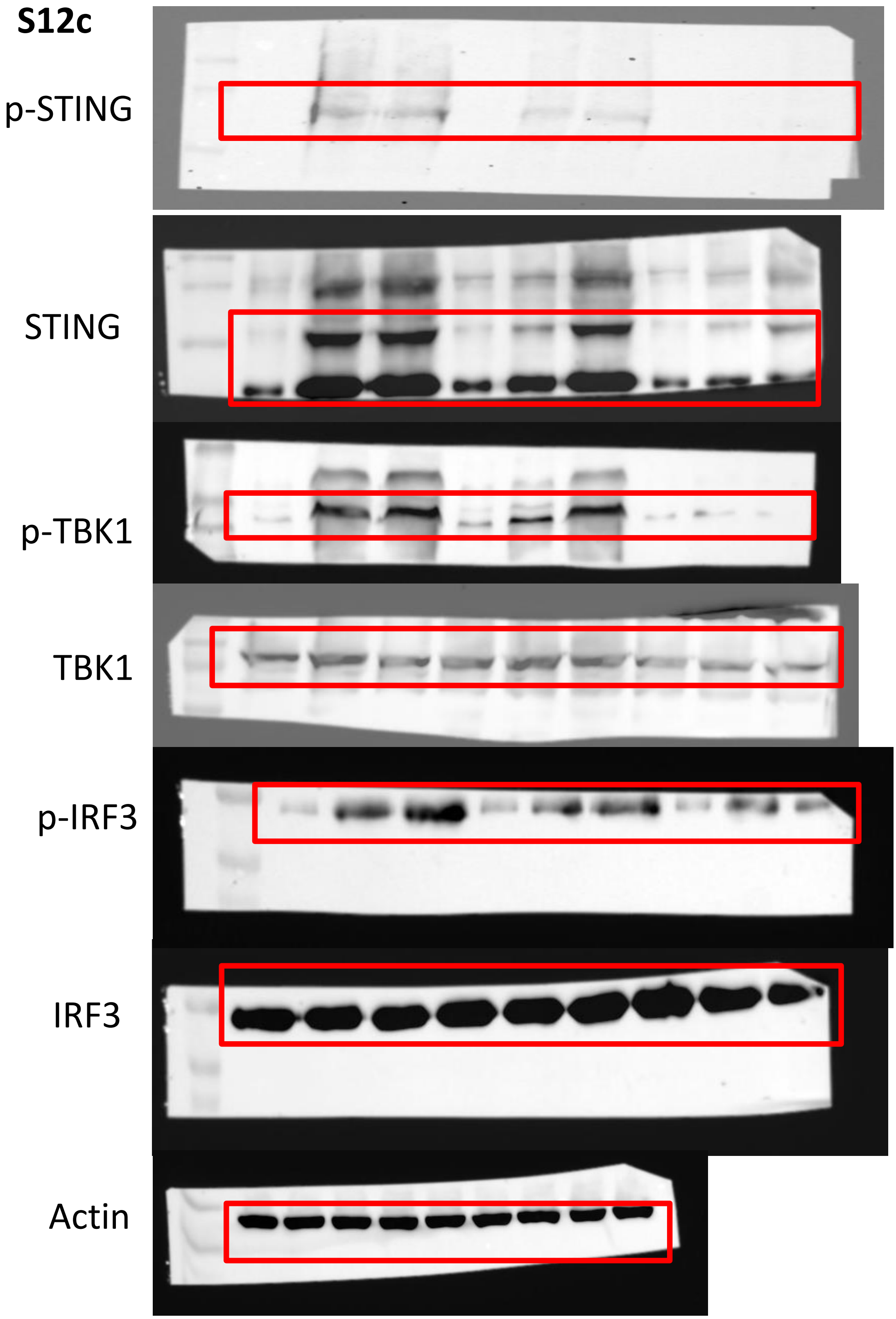

Supplement: Supplementary file 1 — Supplementary Information [file 41467_2018_7425_MOESM1_ESM.pdf]
