# Supplementary material for: STING-dependent sensing of self-DNA drives silica-induced lung inflammation
Source: Nat Commun. 2018 Dec 6;9:5226. doi: 10.1038/s41467-018-07425-1 (PMC6283886; doi:10.1038/s41467-018-07425-1)
Supplement: Supplementary file 3 — Description of Additional Supplementary Files [file 41467_2018_7425_MOESM3_ESM.pdf]

Description of additional supplementary files

Movie 1- Non exposed macrophages

Movie 2- WT BMDMs silica
