# Supplementary material for: STING-dependent sensing of self-DNA drives silica-induced lung inflammation
Source: Nat Commun. 2018 Dec 6;9:5226. doi: 10.1038/s41467-018-07425-1 (PMC6283886; doi:10.1038/s41467-018-07425-1)
Supplement: Supplementary file 27 — Reporting Summary [file 41467_2018_7425_MOESM27_ESM.pdf]

nature research

Corresponding author(s): *Double-blind peer review submissions: write DBPR and your manuscript number here instead of author names.*

Reporting Summary

Nature Research wishes to improve the reproducibility of the work that we publish. This form provides structure for consistency and transparency in reporting. For further information on Nature Research policies, see Authors & Referees and the Editorial Policy Checklist.

Statistical parameters

When statistical analyses are reported, confirm that the following items are present in the relevant location (e.g. figure legend, table legend, main text, or Methods section).

n/a	Confirmed	
☐	☒	The exact sample size (n) for each experimental group/condition, given as a discrete number and unit of measurement
☐	☒	An indication of whether measurements were taken from distinct samples or whether the same sample was measured repeatedly
☐	☒	The statistical test(s) used AND whether they are one- or two-sided<br>*Only common tests should be described solely by name; describe more complex techniques in the Methods section.*
☐	☒	A description of all covariates tested
☐	☒	A description of any assumptions or corrections, such as tests of normality and adjustment for multiple comparisons
☐	☒	A full description of the statistics including central tendency (e.g. means) or other basic estimates (e.g. regression coefficient) AND variation (e.g. standard deviation) or associated estimates of uncertainty (e.g. confidence intervals)
☒	☐	For null hypothesis testing, the test statistic (e.g. F, t, r) with confidence intervals, effect sizes, degrees of freedom and P value noted<br>*Give P values as exact values whenever suitable.*
☒	☐	For Bayesian analysis, information on the choice of priors and Markov chain Monte Carlo settings
☒	☐	For hierarchical and complex designs, identification of the appropriate level for tests and full reporting of outcomes
☒	☐	Estimates of effect sizes (e.g. Cohen's d, Pearson's r), indicating how they were calculated
☐	☒	Clearly defined error bars<br>*State explicitly what error bars represent (e.g. SD, SE, CI)*

Our web collection on statistics for biologists may be useful.

Software and code

Policy information about availability of computer code

Data collection	no computer code used
Data analysis	Prism version 6 software (GraphPad) was used to generate graphs and perform statistical analyses.<br>FlowJo software (Tree Star) version7.6.5 for FACS analyses.<br>Genesys software for western blot analyses.<br>LSM Image Browser (Zeiss) sofware was used for immunofluorescence confocal microscopy analysis.<br>AxioVision Real (Zeiss) 4.9.1 software was used for in vitro live microscopy.

For manuscripts utilizing custom algorithms or software that are central to the research but not yet described in published literature, software must be made available to editors/reviewers upon request. We strongly encourage code deposition in a community repository (e.g. GitHub). See the Nature Research guidelines for submitting code & software for further information.

Data

Policy information about availability of data

All manuscripts must include a data availability statement. This statement should provide the following information, where applicable:

- Accession codes, unique identifiers, or web links for publicly available datasets
- A list of figures that have associated raw data
- A description of any restrictions on data availability

All Figures have associated raw data.

Field-specific reporting

Please select the best fit for your research. If you are not sure, read the appropriate sections before making your selection.

☒ Life sciences ☐ Behavioural & social sciences ☐ Ecological, evolutionary & environmental sciences

For a reference copy of the document with all sections, see nature.com/authors/policies/ReportingSummary-flat.pdf

Life sciences study design

All studies must disclose on these points even when the disclosure is negative.

Sample size	Sample size was chosen to give enough statistical power while using as few animals as possible. For mouse experiments, sample sizes were chosen based on the basis of our previous publications without prior power analysis. Exact numbers of animals used in individual experiments are indicated in the figure legends.
Data exclusions	No data were excluded
Replication	All experiments were reliably reproduced and results are presented as mean +/- SEM as indicated in the figure legends. The results presented have been successfully replicated in at least two independent experiments, with sufficient independent samples. We have carefully reported the experimental conditions in the Online Methods and indicated precisely the nature of replicates in the figure legends.
Randomization	All mice were age and sex-matched and then randomized into the different groups.
Blinding	The investigators were not blinded to group allocation during experiments. Conclusions were made based on quantitative parameters and statistical significance of the data, and thus on experimental observations, independent of blinding.

Reporting for specific materials, systems and methods

Materials & experimental systems

n/a	Involved in the study
☐	☒ Unique biological materials
☐	☒ Antibodies
☒	☐ Eukaryotic cell lines
☒	☐ Palaeontology
☐	☒ Animals and other organisms
☒	☐ Human research participants

Methods

n/a	Involved in the study
☒	☐ ChIP-seq
☐	☒ Flow cytometry
☒	☐ MRI-based neuroimaging

Unique biological materials

Policy information about availability of materials

Obtaining unique materials	All unique materials are readily available from the authors.

Antibodies

Antibodies used	All informations of antibodies used in the study were listed in method section, for immunofluorescence, immunoblots and flow cytometry. STING-specific and pSTING-specific antibodies were validated in STING-deficient mice.

Primary antibodies used were from rabbit anti-STING (#ab92605; Abcam), rabbit anti-TBK1/NAK (#D1B4; Cell Signaling), rabbit anti-IRF-3 (#D83B9; Cell Signaling), rabbit anti-MLKL (#MABC604; Merck), rabbit anti-GSDMD (#ab209845; Abcam), rabbit anti-NLRP3 (#15101; Cell Signaling), rabbit anti-phospho-STING (Ser366; #19781; Cell Signaling), mouse anti-phospho-MLKL (Ser345; #MABC1158; Merck), rabbit anti-Phospho-TBK1/NAK (Ser172; #D52C2; Cell Signaling), rabbit anti-Phospho-IRF-3 (Ser396; #4D4G; Cell Signaling), goat anti-mouse alpha smooth muscle actin (#ab21027; Abcam) and rabbit anti-human beta actin (#ab227387; Abcam).
fluorochrome-conjugated antibodies against mouse CD45-AlexaFluor 700 (1:100; 30-F11), CD11b-BV421 (1:300; M1/70), CD11c-APC-Cy7 (1:400; N418), F4/80-PE-Cy7 (1:400; BM8), Ly6G-BV605 (1:100; 1A8), Ly6C-FITC (1:200; HK1.4), CD4-APC (1:200; GK1.5), TCRβ-BV510 (H57-597 and CD170-PerCp-Cy5.5 (1:100; E50-2440). To verify BMDM and BMDC culture purity and activation, cells were stained as above and using in addition antibodies to mouse MHCII-BV785 (1:300; M5/114), CD40-PE (1:100; 3/23), and CD86-BV605 (1:300; GL-1). All antibodies were from BioLegend except CD170-PerCp-Cy5.5 (BDBiosciences®).

Validation

All these antibodies were validated by manufacturers and largely described in the litterature. Anti-Tmem173 Abs were also validated in Western Blot assays using STING KO mice.

Animals and other organisms

Policy information about studies involving animals; ARRIVE guidelines recommended for reporting animal research

Laboratory animals

C57BL/6J (wild type) mice were purchased from Janvier Labs. Mice deficient for STING (STING-/-) (Ahn et al., 2012), cGAS (cGAS-/-)(Li et al., 2013), IFNAR (IFNAR-/-)(Muller et al., 1994), TLR9 (TLR9-/-)(Hemmi et al., 2000), Gasdermin D (GSDMD-/-) (Schneider et al., 2017) and IL-1R1 (IL-1R1-/-)(Labow et al., 1997) were bred in our specific pathogen free animal facility at CNRS (TAAM UPS44, Orleans, France). For experiments, adult (8-12 week old) animals were kept in ventilated cages in our animal unit and monitored daily. All animal experiments complied with the French Government's animal experiment regulations and were approved by the "Ethics Committee for Animal Experimentation of CNRS Campus Orleans" (CCO) under number CLE CCO 2015-1087. All animals were age- and sex-matched, and then randomized into the different groups. Exact numbers of animals used in individual experiments are indicated in the figure legends. No statistical methods were used to predetermine sample size. The investigators were not blinded to allocation during experiments and outcome assessment.

Wild animals

The study did not involve wild animals.

Field-collected samples

The study did not involve samples collected from the fields.

Flow Cytometry

Plots

Confirm that:

☒ The axis labels state the marker and fluorochrome used (e.g. CD4-FITC).

☒ The axis scales are clearly visible. Include numbers along axes only for bottom left plot of group (a 'group' is an analysis of identical markers).

☒ All plots are contour plots with outliers or pseudocolor plots.

☒ A numerical value for number of cells or percentage (with statistics) is provided.

Methodology

Sample preparation

described in Material and Methods in detail

Instrument

described in Material and Methods in detail

Software

described in Material and Methods in detail

Cell population abundance

No cell sorting

Gating strategy

The gating strategy is shown in the Figures and further described in the supplementary Figures.

☒ Tick this box to confirm that a figure exemplifying the gating strategy is provided in the Supplementary Information.
